# Placental sufficiency is relational: a demand–supply hypothesis for divergent maternal and fetal expressions of gestational disease

**DOI:** 10.3389/fphys.2026.1963994

**Published:** 2026-10-19

**Authors:** Maria Cezara Mureşan, Marius Bogdan Mureşan

**Affiliations:** 1 Department of Obstetrics and Gynecology, “Victor Babeş” University of Medicine and Pharmacy, Timişoara, Romania; 2 Fetal Medicine Center, Medicis/MedLife Hospital, Timişoara, Romania

**Keywords:** fetal compromise, fetal growth restriction, fetal oxygen demand, maternoplacental supply, placental growth factor (PIGF), placental insufficiency, pre-eclampsia, reference endpoint

## Abstract

Placental insufficiency is usually identified indirectly—through the maternal syndrome, the growth restriction it causes or lesions found after delivery. None of these defines the placenta’s oxygen-transfer function, and each can be absent while fetal support is already inadequate. We propose that this function is relational: it should be defined by the balance between the fetal oxygen requirement, *C*_o_(*t*), and the effective capacity of the integrated maternal–placental–fetal chain to supply oxygen, *O*_s_(*t*), assessed at the fetal receiving end. Their difference, Δ(*t*) = *C*_o_(*t*) − *O*_s_(*t*), is a latent state rather than a measurable quantity; a positive imbalance denotes insufficiency of the integrated transfer relation and does not by itself identify the limiting component or imply intrinsic placental pathology, which a separate aetiological step must establish. Insufficiency is reached by supply-driven, demand-driven or, most commonly, mixed trajectories—the demand-driven route arising without a previously detectable placental abnormality. We distinguish this latent imbalance from placental signalling, which may reflect or mediate it but is not identical to it, and from its host-dependent clinical expressions. For placental growth factor (PlGF), we advance a falsifiable hypothesis: under a sustained positive imbalance, the highly oxidative placenta may shift towards oxygen-sparing metabolism, and the associated hypoxia-responsive and integrated stress-response signalling may lower PlGF, so that a gestational-age-inappropriate PlGF decline behaves as a delayed signal of sustained mismatch rather than a direct measure of fetal demand. Because the maternal syndrome is a host-dependent expression of the same signalling, its severity is only incompletely coupled to the fetal consequence, and fetal compromise can occur during relative maternal silence. We therefore argue that the reference endpoint for placental oxygen-transfer function should shift from the maternal syndrome to the adequacy of fetal support, with maternal and fetal morbidity remaining co-equal outcomes. The framework yields predictions on biomarker trajectories and maternal–fetal divergence, and explicit, multi-level conditions under which it would fail.

## 1 Introduction: placental insufficiency and the single-endpoint problem

How should placental function be defined: by the structure of the organ, by the maternal syndrome it may generate, or by its capacity to meet fetal requirements? In practice, the three are frequently treated as interchangeable, and the answer defaults to whichever is easiest to observe. Placental insufficiency is inferred from abnormal uterine artery Doppler, from fetal growth restriction, from pre-eclampsia or from placental histopathology after birth. Each of these is informative, but each identifies a cause or an expression of placental disease rather than the function itself, and each can be absent in a pregnancy whose capacity for fetal support is already marginal.

The most consequential of these proxies is the maternal syndrome. Screening and surveillance for late-gestation placental disease have been organised, with considerable success, around the prediction of pre-eclampsia: contemporary competing-risks models calibrate the timing of the maternal phenotype against gestational age with real accuracy (Wright et al., 2020), and international guidance recognises angiogenic imbalance as a marker of uteroplacental dysfunction, yet retains it within a diagnostic frame anchored on maternal hypertension (Magee et al., 2022). This organisation is not a mistake; on its own target, it performs well. The difficulty lies one level deeper, in the choice of target. A latent state that has more than one expression is being read through only one of them.

We call this the single-endpoint problem, and it must be stated precisely to avoid caricaturing clinical practice. Obstetrics does not monitor the placenta through pre-eclampsia alone: fetal growth assessment, umbilical and cerebral artery Doppler, maternal perception of fetal movements, stillbirth-prediction models and intrapartum cardiotocography all address the fetal axis directly. The problem is narrower. It concerns the tools and biomarkers that are built or calibrated on a maternal endpoint—most importantly the angiogenic markers and competing-risks models developed to predict pre-eclampsia—when their output is implicitly read as evidence of fetal safety. Such an instrument reports, by construction, on how strongly one actor responds. Because it is calibrated on a maternal endpoint, it is not designed to distinguish reliably between absence of the underlying imbalance and its presence with limited maternal expression. In the latter situation, a low estimated maternal risk may provide relative reassurance without establishing adequate fetal support. The result is a blind region—a relative maternal silence—in which fetal support may be failing while the maternal readout remains unremarkable, and the absence of a maternal phenotype may be over-interpreted as reassurance about fetal support in the absence of direct fetal assessment.

Resolving this requires a definition of placental function that is not tied to any single expression. We propose a relational one. Throughout this article, placental sufficiency denotes the *in vivo* capacity of the maternal–placental–fetal oxygen-support system, operating through the placenta, to meet fetal requirements. It is therefore a functional and relational property, not a claim that the limiting lesion is necessarily intrinsic to the placenta; we reserve ‘localised uteroplacental dysfunction’ for situations in which the bottleneck can be assigned specifically to the uteroplacental unit. Placental sufficiency is thus defined by the balance between fetal oxygen demand and the effective capacity of that system to supply it; insufficiency is the state in which demand approaches or exceeds that capacity, whatever its cause and whichever actor comes to express it. The remainder of this article develops the model and its consequences: the demand–supply balance and its behaviour over gestation and in labour (Section 2); the trajectories by which relational insufficiency can arise (Section 3); the relationship between the latent imbalance and placental signalling, including a specific, falsifiable mechanistic hypothesis for placental growth factor (PlGF) (Section 4); the divergence of maternal and fetal expressions (Section 5); the consequent shift in the reference endpoint of placental oxygen-transfer function (Section 6); the model’s relationship to existing frameworks (Section 7); and its testable predictions and the conditions that would falsify it (Section 8), before its limitations and scope (Sections 9 and 10).

Stated in full, the hypothesis is as follows. Placental insufficiency is not solely an intrinsic property of placental structure, but a relational state arising when fetal oxygen demand approaches or exceeds effective system-level oxygen supply. Placental signalling may reflect or mediate this imbalance, while its maternal and fetal expressions depend on distinct host susceptibilities and may therefore diverge. Pre-eclampsia is, on this account, a host-dependent maternal expression of placental signalling, whereas fetal compromise may arise when oxygen supply becomes inadequate relative to fetal requirement, including during relative maternal silence.

## 2 Placental sufficiency as a relational state

### 2.1 The demand–supply balance

Placental insufficiency is commonly inferred from its presumed causes or clinical consequences. Abnormal uterine artery Doppler findings, fetal growth restriction, pre-eclampsia and placental histopathology each identify important components of placental disease, but none defines placental function itself. A placenta can be structurally abnormal yet continue to meet fetal requirements for a period of time; conversely, apparently normal fetal growth, maternal normotension and unremarkable uteroplacental Doppler findings do not establish that the capacity for fetal support remains adequate. The functional question is therefore relational: adequate for what level of fetal requirement, and under what physiological conditions?

We define sufficiency through the balance between fetal oxygen demand and the effective capacity of the maternal–placental–fetal chain to supply oxygen. Let *C*_o_(*t*) denote the fetal oxygen requirement at time *t*—the demand the fetus would have in reference physiology, conditioned on gestational age, fetal mass and activity, before any adaptation—and *O*_s_(*t*) the effective system-level capacity to supply oxygen to the fetus at that time; the subscripts are retained throughout, in text, equations, tables and figures (**Box 1**). *C*_o_ and *O*_s_ are latent physiological rates conceived on a common oxygen scale (for example, millilitres of O_2_ per minute). Because current proxies do not identify them directly in those units, Δ and the cumulative-exposure term *B* (below) remain latent physiological constructs rather than calibrated bedside quantities. Their difference is represented as:


$$\Delta \left(t\right)\;=C_{o}\left(t\right)\;-O_{s}\left(t\right).$$

Here, *O*_s_(*t*) is capacity rather than observed oxygen transfer. Defining supply by the amount actually transferred would risk tautology, because observed transfer is constrained by consumption and may fall when fetal metabolism is suppressed. Effective supply capacity integrates maternal oxygen content, uterine blood flow, intervillous perfusion, placental exchange area and diffusion, placental oxygen consumption and umbilical blood flow. Fetal haemoglobin concentration and the position of the oxygen-dissociation curve subsequently modify the amount of oxygen that can be carried and utilised by the fetus. HbF-related affinity facilitates placental oxygen loading at low fetal PO_2_, but net transport and tissue unloading depend jointly on haemoglobin concentration, saturation, pH/PCO_2_, flow and extraction (Enoki et al., 1972; Wilkening et al., 1988; Carter, 2015); these are therefore treated as fetal-side modifiers of system-level oxygen transport, not as a fixed multiplier of *O*_s_ or as a means of localising a uteroplacental lesion. No single current clinical measurement captures all these components.

Box 1 **Symbols used throughout.**

**Table:** 

Symbol	Meaning	Status
*C*_0_(*t*)	Unadapted fetal oxygen requirement (counterfactual, reference physiology)	Latent (counterfactual)
*C*_a_(*t*)	Realised fetal oxygen consumption after adaptation	Latent
*O*_s_(*t*)	Effective system-level oxygen-supply capacity	Latent
Δ(*t*) = *C*_0_ − *O*_s_	Instantaneous imbalance	Latent (ordinal)
*R* = (*O*_s_ − *C*_0_)/*C*₀	Fractional reserve (re-expression of the balance)	Latent; not directly estimated
*B*(*t*)	Cumulative exposure to positive imbalance	Latent; only downstream consequences (BDecf, lactate), not proxies
*V*_m_	Maternal response-modifying function	Conceptual, prespecified
*V*_f_	Fetal response-modifying function	Conceptual, prespecified

Demand must be handled with the same care, and two quantities must be distinguished. *C*_o_(*t*) is the unadapted requirement defined above. *C*_a_(*t*) is the fetus’s realised oxygen consumption after adaptation—which it can transiently lower by redistributing cardiac output, reducing activity and slowing growth. The imbalance is defined on the requirement, Δ(*t*) = *C*_o_(*t*) − *O*_s_(*t*), not on realised consumption: were it defined on *C*_a_, a fetus compensating pathologically could appear to have returned Δ towards zero precisely because it was failing. Fetal adaptation therefore lowers *C*_a_ and buys time without resolving Δ. The instantaneous imbalance does not capture the duration or recurrence of exposure. We therefore define *B*(*t*) = ∫_o_*^t^* [*C*_o_(*τ*) − *O*_s_(*τ*)]_+_ d*τ* as cumulative exposure to positive imbalance. Because oxygen is not stored in a physiologically meaningful reservoir, *B*(*t*) does not represent depletion of an oxygen stock or fetal reserve; it summarises the magnitude and duration of unmet requirement. Its metabolic consequences depend additionally on fetal compensatory capacity, the temporal pattern of exposure and the opportunity for recovery between episodes. Cord-blood lactate and base deficit are downstream integrative indicators of metabolic cost, not direct measurements of *B*(*t*).

Because *O*_s_(*t*) integrates maternal, uteroplacental and umbilical components, Δ(*t*) is a property of the integrated maternal–placental–fetal oxygen-transfer relation, assessed at its fetal receiving end. Relational placental oxygen-transfer insufficiency is present when this integrated capacity is inadequate for the current fetal requirement. This functional designation does not by itself localise the limiting component or imply intrinsic placental pathology. A separate aetiological step distinguishes maternal/pre-placental limitations (for example, maternal hypoxaemia, anaemia or cardiovascular limitation), uteroplacental, umbilical (for example, cord compression) and mixed limitations; empirical testing must establish that localisation rather than assume it.

Similarly, Δ(*t*) is not proposed as a directly measurable biomarker or a dimensionally calibrated bedside quantity. It represents a latent state. When Δ < 0, effective capacity exceeds current demand and a functional reserve exists. When Δ ≈ 0, demand approaches the effective ceiling of supply and reserve is limited. When Δ > 0, current demand is not fully met. The biological consequences of a positive imbalance depend not only on its magnitude but also on its duration, frequency and temporal pattern. Brief positive excursions may be compensated; sustained or repeatedly induced positive imbalance increases the probability that fetal compensatory mechanisms will be exhausted.

### 2.1.1 The identification problem for *C*_o_ and a fractional-reserve formulation

Δ(*t*) is a definitional physiological state variable, not the proposed clinical biomarker. A positive Δ denotes that the effective oxygen-supply capacity of the integrated maternal–placental–fetal transfer chain, assessed at its fetal receiving end, is insufficient for the unadapted fetal requirement. The empirical objective is therefore not to recover a numerical Δ at the bedside, but to identify pregnancies likely to have crossed into Δ > 0 through prespecified observable proxies and objective fetal endpoints. This establishes relational oxygen-transfer insufficiency; it does not, by itself, localise the bottleneck or imply intrinsic placental pathology.

Two distinct questions follow and must be kept apart. First, is the supply chain adequate for the fetal requirement?—The question Δ = *C*_o_ − *O*_s_ is built to answer. Second, where is the limitation that reduced supply: maternal/pre-placental, uteroplacental, umbilical or mixed? *O*_s_ integrates the whole maternal–placental–fetal chain evaluated at the fetal receiving end; the placenta is the obligate interface through which oxygen reaches the fetus, but Δ > 0 does not by itself identify the defective component. This does not weaken the relational definition; it is precisely its meaning: ‘placental sufficiency’ describes the adequacy of the placenta in the context of maternal supply and fetal requirement, not proof of an intrinsic lesion of placental tissue. A positive Δ, moreover, need not imply overt tissue hypoxia at every moment: fetal extraction, redistribution and a reduction in realised consumption (*C*_a_) can temporise its expression, which is one reason the *C*_o_/*C*_a_ distinction is retained.

*C*_o_ itself is counterfactual—the requirement the fetus *would* have in reference physiology—and cannot be recovered by any single measurement in a living pregnancy (**Table 1**); no current *in vivo* measurement directly recovers *C*_o_, *O*_s_, Δ or *B*. Observable analogues nonetheless bound the balance from the measured side: umbilical venous oxygen delivery and the fetal oxygen extraction fraction, accessible in principle through combined Doppler and Fick estimation (Acharya and Sitras, 2009; Cetin et al., 2020) and, increasingly, through fetal MRI oximetry (Rodríguez-Soto et al., 2018; Saini et al., 2020); these are component indicators of realised oxygen transport, not measurements of the counterfactual requirement. *R*(*t*) = (*O*_s_ − *C*_o_)/*C*_o_ is a fractional conceptual re-expression of the same latent balance—*R* > 0 preserved reserve, *R* → 0 the approach to the ceiling, *R* < 0 a sustained deficit. It does not make *C*_o_, *O*_s_, Δ or their localisation observable. Proxy-based analyses aim to identify pregnancies likely to have crossed into Δ > 0; they do not estimate *R* directly.

**Table 1:** Latent constructs of the model, candidate proxies and what each proxy can and cannot measure.

A Proxy, analytic role, timing and what it measures.				
Latent construct	Candidate proxy	Analytic role	Timing	What it measures
*C*_o_—unadapted fetal oxygen requirement	Gestational age, estimated fetal weight	Adjustment covariate/eligibility (expected requirement)	T1–T3	Expected requirement in reference physiology
*O*_s_—system supply capacity	Uterine/umbilical Doppler; maternal SpO_2_ and haemoglobin; placental T2*	Supply exposure indicator	T1–T3, serial	Flow and impedance; maternal oxygen content; bulk placental oxygenation
Δ and cumulative exposure *B*(*t*)	None direct (inferred)	Latent target construct	Inferred only	–
Fetal consequence of Δ > 0	Cord metabolic acidaemia, encephalopathy, cardiotocography	Outcome	At/after birth	Downstream hypoxic-ischaemic injury
Placental metabolic phase	Placental T2*; birth lactate and metabolomics	Mediator	*Ex vivo*/imaging; birth	Bulk oxygenation; end-state metabolites
PlGF signal	Maternal free and total PlGF, sFlt-1 (MoM, trajectory)	Primary predictor (signal)	Serial (32/36 weeks)	Circulating angiogenic state

B Dominant measurement error, what it cannot identify and expected direction under competing models.				
Latent construct	Dominant measurement error	What it cannot identify	Expected direction under competing models	
*C*_o_—unadapted fetal oxygen requirement	GA misdating and EFW error; largely non-differential (towards the null)	The individual metabolic set-point; may already be altered by disease	Rises with fetal mass under all models (not discriminating)	
*O*_s_—system supply capacity	Vessel-diameter, angle and timing error; largely non-differential (towards the null)	Actual oxygen transfer; the integrated capacity as a whole	Relational model: no universal direction of *O*_s_ is assumed; adequacy depends on supply relative to requirement. Supply-predominant trajectory: lower or blunted supply proxies. Fixed-lesion rival: early and persistent abnormality	
Δ and cumulative exposure *B*(*t*)	Not measured by any assay	The imbalance itself; only its consequences are observable. Cord lactate and base deficit are downstream metabolic consequences, *not* proxies of *B*(*t*)	Not directly observable under any model	
Fetal consequence of Δ > 0	Ascertainment- and adjudication-dependent; misclassification towards the null unless linked to management	Placental versus extraplacental cause, without adjudication	Common to all models; adjudication distinguishes cause	
Placental metabolic phase	Bulk spatial averaging (imaging); end-state-only metabolites; towards the null	The longitudinal sequence; fetal-capillary versus trophoblast	Biphasic: late metabolic shift. Fixed-lesion: early/static	
PlGF signal	Assay/matrix and gestational-timing variation; MoM standardisation reduces but does not remove it	Production versus release versus sequestration from free PlGF alone; it is not an O_2_ sensor	Biphasic (production): late concordant free+total fall. Sequestration: disproportionate free fall. Fixed-lesion: early, static low PlGF	

This definition has two consequences. First, placental sufficiency is not an immutable property of placental structure. The same organ may be sufficient for a smaller fetus at an earlier gestational age and insufficient for a larger fetus later in gestation. Second, a quantitatively unchanged supply can become functionally inadequate as demand rises. Thus, ‘relative supply deficit’ does not necessarily mean that maternal oxygen delivery or placental transfer capacity has abruptly fallen. Supply may remain quantitatively stable while becoming inadequate relative to the requirement it must serve.

Oxygen is treated as the acute limiting currency most directly linked to fetal compromise—time-critical, not storable in meaningful quantities, and repeatedly challenged during labour—not as the exclusive metabolic determinant of placental function. Substrate availability, maternal metabolism and the placenta’s own metabolic cost may act as upstream determinants of the balance or as parallel axes; the model neither denies them nor absorbs them, and it does not become a theory of all placental substrates. The oxygen axis provides the clearest and most acutely decisive physiological route linking a supply–demand imbalance to fetal injury.

### 2.2 Functional domains and temporal exposure

The three domains of Δ should not be interpreted as sharply separated biological compartments. Near the boundary between reserve and insufficiency, small changes in maternal oxygenation, uterine perfusion, fetal activity or contractile stress may change the instantaneous balance. A fetus may therefore remain clinically compensated despite recurrent episodes in which effective supply temporarily fails to meet demand.

Fetal adaptation acts on realised consumption, *C*_a_, not on the underlying requirement, *C*_o_. Redistribution of cardiac output and reduced activity may lower realised oxygen consumption, while slowing growth may limit its further rise; neither reduces the requirement the fetus would have in reference physiology, and neither by itself resolves Δ. Fetal growth restriction can consequently be an adaptation to, or consequence of, impaired supply rather than a necessary definition of relational insufficiency. An appropriately grown fetus may have experienced no previous deficit, may have compensated without measurable growth restriction or may have reached a demand level that only recently exceeds supply. Size alone cannot distinguish these possibilities.

Time is therefore intrinsic to the model. An early, severe reduction in *O*_s_ may produce prolonged positive imbalance, impaired growth and overt Doppler abnormalities. At term, a smaller mismatch may remain clinically silent until fetal demand rises, the per-mass supply margin narrows or a physiological stressor transiently lowers supply. These trajectories can converge on a similar instantaneous Δ > 0 despite different placental histories.

### 2.3 Labour as a physiological stress test

Labour makes the relational nature of placental sufficiency particularly visible (**Figure 1**). Uterine contractions intermittently reduce uteroplacental perfusion and gas exchange; physiological accounts estimate a substantial reduction *in utero*placental perfusion during contractions (Turner et al., 2020). In a fetus entering labour with ample reserve, oxygenation and acid–base status recover between contractions. When pre-labour demand is already close to effective supply capacity, the same contraction pattern can repeatedly move the system into a positive imbalance. Recovery becomes incomplete, myocardial and metabolic reserves are consumed, and compensatory fetal heart-rate responses may ultimately fail (Turner et al., 2020). This intrapartum unmasking has recently been described explicitly as a ‘relative uteroplacental insufficiency of labour’, in which contractions alter the fetal oxygen demand–supply equation even when antenatal reserve appeared adequate (Ghi et al., 2024); the present model treats that phenomenon as the labour-specific case of a general relational insufficiency that also operates antenatally.

**Figure 1:**
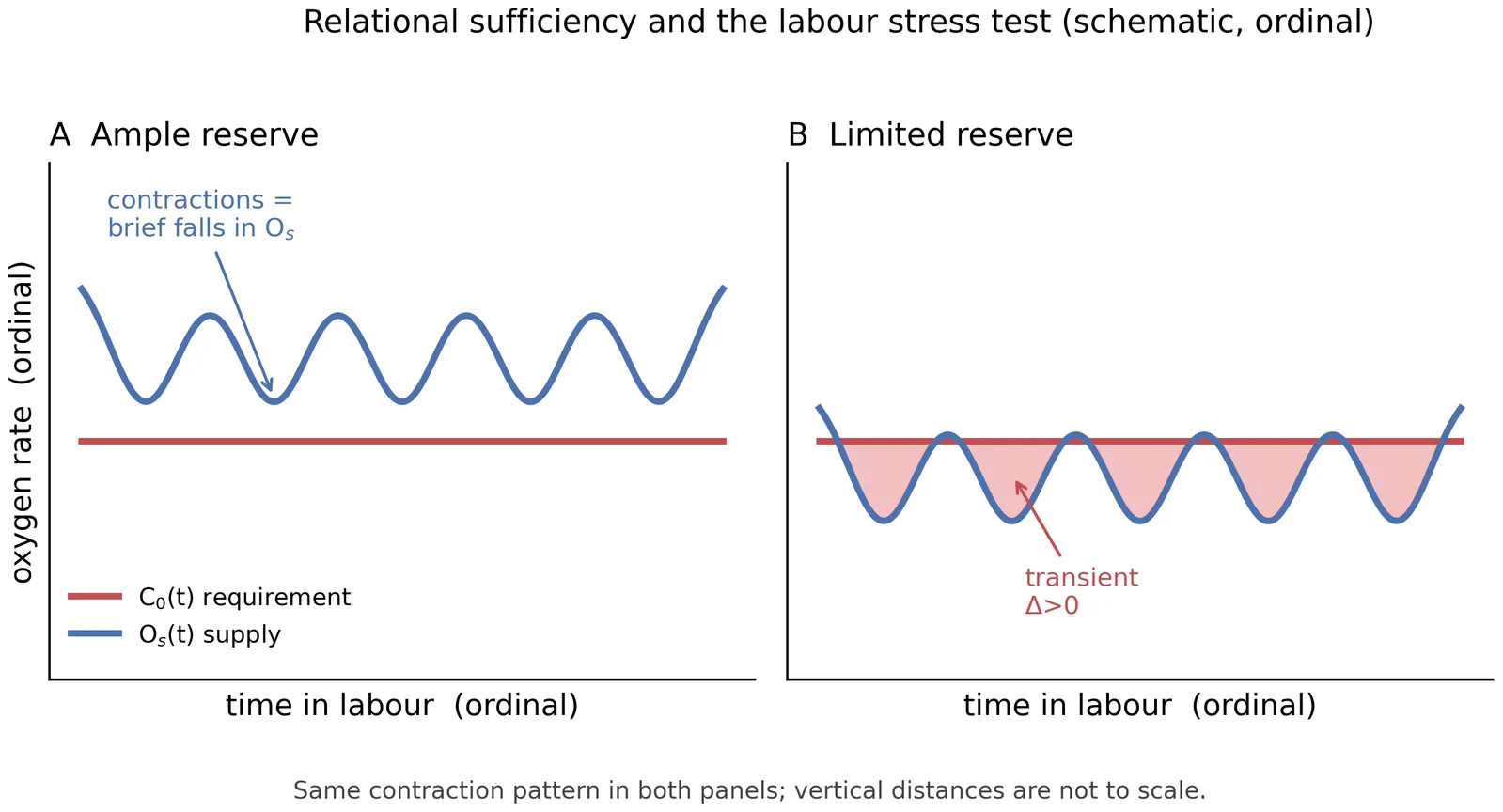
Relational placental sufficiency and the labour stress test (schematic, ordinal). The panels compare a pregnancy with ample reserve **(A)** and a susceptible pregnancy with limited reserve **(B)** under the same contraction pattern. In each, the baseline fetal oxygen requirement *C*_o_(*t*) is held constant across the labour panel, and effective system oxygen-supply capacity *O*_s_(*t*) is constant between contractions; each contraction is shown only as a transient downward deflection in *O*_s_(*t*)—a brief fall in supply, not a rise in demand. Functional reserve is present when Δ(*t*) = *C*_o_(*t*) − *O*_s_(*t*) < 0; the same contraction-related deflections leave Δ < 0 where the initial margin is wide **(A)** but drive transient Δ > 0 where it is limited **(B)**. Curves are schematic and ordinal; vertical distances and slopes are not quantitatively scaled, and no numerical *C*_o_/*O*_s_/Δ trajectory is implied. Published flow, oxygen-delivery and uptake values are used only as separate anchors for measurable components in the text, not combined into a single quantitative curve.

Labour is therefore not necessarily the origin of placental insufficiency. It may be the first condition that reveals a limited reserve that was clinically silent at rest. This distinction explains why intrapartum compromise can occur in pregnancies classified as low risk immediately before labour, and why intrapartum Doppler indices can carry prognostic information for compromise in fetuses not previously identified as growth-restricted (Packet et al., 2025). It also supports treating operative delivery for suspected fetal compromise as a management-dependent proxy, while cord metabolic acidaemia, neonatal encephalopathy and intrapartum or antepartum death are less management-dependent—though not placenta-specific—endpoints that require adjudication against acute extraplacental causes.

## 3 Trajectories to relational placental insufficiency

Relational insufficiency is a single state—a sustained Δ > 0—that can be approached along different trajectories. Because Δ(*t*) = *C*_o_(*t*) − *O*_s_(*t*), its rate of change is governed by the two derivatives,


$$d\Delta /dt=\;dC_{o}/dt-\;dO_{s}/dt,$$

so a positive imbalance can become predominant either because effective supply falls or plateaus early (a supply-driven trajectory), because rising fetal demand overtakes an initially adequate capacity (a demand-driven trajectory), or, most commonly, through a mixture of the two. These are archetypes of how the balance tips (**Figure 2A–D**), not two separate diseases or two separate mechanisms: at the moment of insufficiency, both describe the same relation, *C*_o_ ≥ *O*_s_, and differ only in which derivative drove the system there and in when it did so. Presenting them as trajectory archetypes rather than distinct aetiologies avoids implying that a clinician must decide *a priori* which side ‘caused’ the imbalance, and it accommodates the frequent case in which a modestly reduced ceiling and a rising requirement together produce insufficiency that neither would have produced alone.

### 3.1 The supply-driven trajectory

The established trajectory begins with reduced supply capacity. Defective trophoblast invasion, incomplete spiral artery remodelling, placental vascular maldevelopment, infarction, inflammation or other placental lesions can lower the ceiling of *O*_s_(*t*). As fetal demand rises with gestation, a reduced ceiling is reached earlier. This pathway accounts naturally for early-onset placental disease, fetal growth restriction, abnormal Doppler findings and many cases of pre-eclampsia (**Figure 2B**, supply-predominant panel).

**Figure 2:**
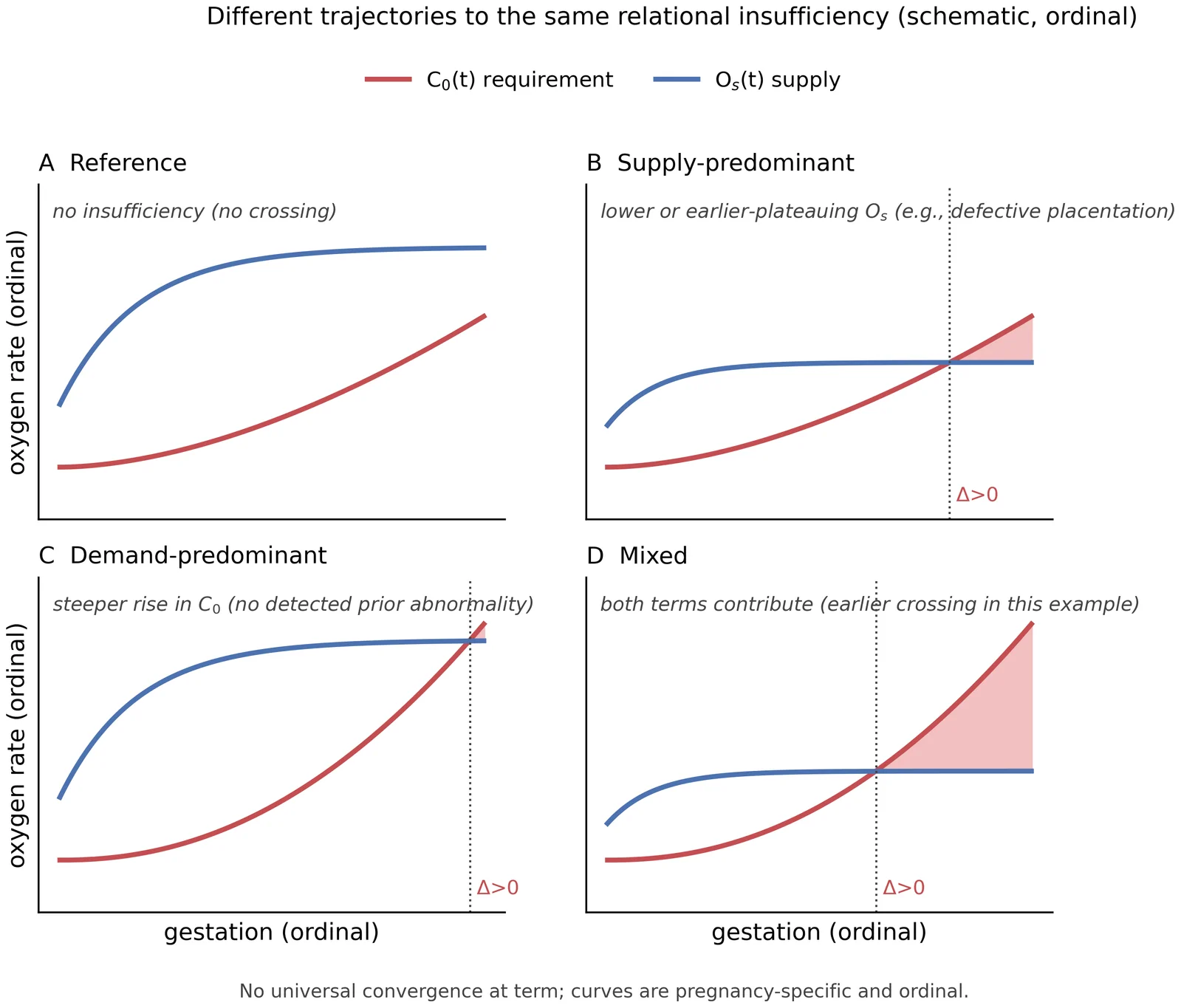
Different trajectories to the same relational insufficiency (schematic, ordinal). Effective supply capacity *O*_s_(*t*) is drawn as a concave curve—rising quickly earlier in gestation and then flattening towards a ceiling—while the unadapted fetal oxygen requirement *C*_o_(*t*) is drawn as a curve that rises slowly at first and accelerates through the second half of pregnancy, easing only slightly near term as weight velocity slows. Four panels show deviations from a reference relation, not separate diseases. **(A)** Reference: *O*_s_(*t*) stays above *C*_o_(*t*) throughout; there is no antenatal crossing. **(B)** Supply-predominant: the same requirement curve meets an *O*_s_(*t*) that starts lower and plateaus earlier and lower (for example, defective placentation, which may both reduce supply and generate a maternal signal; supply may also fall through pre-placental causes); the curves cross. **(C)** Demand-predominant (no detected prior abnormality): *O*_s_(*t*) follows the reference trajectory but *C*_o_(*t*) arcs upward more steeply and overtakes it late, often without a previously detectable structural, growth-related or haemodynamic abnormality—which does not prove normal placental biology. **(D)** Mixed: a lower, earlier-plateauing *O*_s_(*t*) and a steeper *C*_o_(*t*) act together, crossing earlier in this example; probably the most common case. The crossing point is pregnancy-specific and occurs at a different time in each panel; the reference panel does not cross antenatally. Curvature represents qualitative gestational changes in effective oxygen-supply capacity and fetal oxygen requirement. The curves are not measurements of placental mass, fetal weight or blood flow and are not quantitatively scaled.

In this route, defective placentation can have two parallel effects. It can reduce effective transfer capacity, thereby increasing Δ, and it can provoke oxidative and endoplasmic-reticulum stress signalling and the release of factors capable of producing maternal endothelial disease. The placental signal is therefore not itself the cause of the demand–supply imbalance. Rather, defective placentation is a common upstream cause that can both restrict supply and produce a maternal signal. Keeping these levels separate avoids equating the measurable angiogenic phenotype with the latent functional state.

Maternal disease may subsequently feed back onto supply. Hypertension, endothelial dysfunction and systemic organ disease may alter maternal haemodynamics or clinical management and thereby further reduce effective uteroplacental support. Pre-eclampsia can thus be both a host-dependent expression of placental signalling and, once established, a potential amplifier of the underlying supply limitation.

### 3.2 The demand-driven transition to insufficiency (no detected prior abnormality)

A second trajectory is less explicitly represented in current definitions. Placental insufficiency may emerge without a previously detectable structural, growth-related or uteroplacental haemodynamic abnormality. We hypothesise that an initially adequate placenta may cross into functional insufficiency when increasing fetal demand persistently overtakes its effective supply capacity (**Figure 2C**, demand-predominant panel). The term is ‘demand-driven transition to insufficiency’ rather than ‘demand-exceeded sufficiency’: once demand has overtaken capacity, the state is, by definition, no longer sufficiency; what is distinctive is that the rising derivative on the demand side, not a fresh fall in supply, is what tips the balance.

This proposition does not require fetal demand to become pathological. Normal fetal growth progressively increases total oxygen requirement. Nor does it require supply to fall abruptly. A placenta with an initially adequate but finite capacity may approach its ceiling as pregnancy advances. A relative supply deficit emerges when the relation changes from *C*_o_ < *O*_s_ to *C*_o_ ≥ *O*_s_, even if absolute supply remains within a range conventionally regarded as normal.

A rough physiological budget shows the demand-driven trajectory is at least plausible. Doppler/Fick estimates place the oxygen uptake of the appropriately grown human fetus at term at approximately 6.6 mL·kg^-^¹·min^-^¹, or approximately 22 mL/min for a 3.4-kg fetus (Acharya and Sitras, 2009). At term, placental oxygen consumption accounts for approximately 40% of the total oxygen uptake by the gravid uterus (Carter, 2000, 2015). Placental and fetal metabolism therefore share a finite extracted oxygen flux. Human longitudinal Doppler data show that total uterine artery blood flow rises across the second half of pregnancy (from approximately 513 to 970 mL·min^-^¹ between 20 and 38 weeks; Konje et al., 2001), while uterine flow per kilogram of estimated fetal weight falls; absolute umbilical venous flow likewise rises, whereas weight-normalised umbilical venous flow declines after the early second half of pregnancy (Acharya et al., 2005; Flo et al., 2010). A near-delivery cross-sectional comparison reported lower weight-normalised umbilical flow and oxygen transport at full term than preterm (Link et al., 2007), treated here as a supportive anchor rather than a direct estimate of *O*_s_ or *R*. What the model asserts is therefore narrower and conditional: the absolute margin *M* = *O*_s_ − *C*_o_ narrows only when d*O*_s_/d*t* < d*C*_o_/d*t*, and the fractional reserve *R* = (*O*_s_ − *C*_o_)/*C*_o_ narrows only when (1/*O*_s_)d*O*_s_/d*t* < (1/*C*_o_)d*C*_o_/d*t*. There is thus no universal convergence of the two curves; the trajectory is pregnancy-specific, and the margin narrows selectively—in fetuses with an elevated demand derivative or a blunted per-mass supply derivative, or both. The same term oxygen uptake shows no significant difference between uncomplicated labour and pre-labour caesarean delivery (Acharya and Sitras, 2009), consistent with preserved reserve in the normal term fetus; conversely, late-term fetuses with reduced umbilical venous flow, a marked fall in abdominal-circumference centile or cerebral redistribution form a recognised at-risk subgroup (Stampalija et al., 2022). Labour then reveals a limited margin; it does not render the normally reserved term fetus insufficient.

The hypothesis is compatible with, but not identical to, placental maturation or senescence. Senescence, infarction or declining perfusion can reduce *O*_s_; increasing fetal mass and metabolism can increase *C*_o_. Both processes may occur together. The relational model does not require clinicians to decide *a priori* which side initiated the imbalance. It asks whether effective capacity remains adequate for current fetal requirement.

‘No previously detectable abnormality’ must not be interpreted as proof that the placenta was biologically normal. Occult malperfusion, microscopic lesions, reduced exchange efficiency or an intrinsically lower functional ceiling may be disclosed only when demand increases. Distinguishing demand that exceeds an otherwise normal capacity from demand that reveals a pre-existing occult limitation is an empirical question. Importantly, both routes preserve the central definition: functional insufficiency begins when effective supply becomes inadequate relative to demand.

The demand-driven trajectory offers a potential explanation for the vulnerable, appropriately grown fetus at term. Such a fetus may have accumulated sufficient mass to approach its system supply capacity, O_s_, without developing fetal growth restriction or maternal hypertension. At rest, compensation may remain effective. During labour, recurrent reductions in *O*_s_ expose the limited reserve. The first overt clinical event may therefore be intrapartum compromise rather than antenatal growth restriction or pre-eclampsia.

## 4 Placental signalling: readout, mediator or adaptation?

Placental signalling is not identical to the demand–supply imbalance. Circulating biomarkers may reflect placental cellular state, mediate maternal effects or participate in adaptation, but they do not directly measure *C*_o_, *O*_s_ or Δ. This distinction is essential when interpreting the angiogenic axis.

### 4.1 Biological asymmetry between sFlt-1 and PlGF

Soluble fms-like tyrosine kinase-1 (sFlt-1) binds circulating VEGF and PlGF and can produce an antiangiogenic state. Experimental excess sFlt-1 recapitulates important features of the maternal pre-eclamptic syndrome, supporting an active mediating role in endothelial dysfunction. By contrast, reduced circulating PlGF may result from decreased placental production, altered release, increased binding by sFlt-1 or combinations of these processes. Throughout, ‘circulating PlGF’ denotes *free* PlGF unless stated otherwise; free and total PlGF (free plus sFlt-1-bound) diverge according to sFlt-1 binding and to the assay used, a distinction essential to separating production from release and sequestration and one on which the phase-specific prediction below depends (Lecarpentier et al., 2020; Jungelson et al., 2025). The sFlt-1/PlGF ratio is clinically useful because it amplifies angiogenic imbalance, but it can compress two partially distinct biological changes into a single value.

Evidence from matched maternal serum and placental tissue is compatible with this relative asymmetry. In the cited placental-expression study, the increased maternal sFlt-1/PlGF ratio was driven predominantly by increased placental sFlt-1 in pre-eclampsia and by reduced placental PlGF in fetal growth restriction (Gaccioli et al., 2023); this is an observational, context-dependent finding rather than a general rule. Both markers are placentally derived and both associate with maternal and perinatal outcomes. The framework does not assign elevated sFlt-1 uniquely to abnormal placental development, nor reduced PlGF uniquely to a sustained positive demand–supply imbalance. Their longitudinal trajectories should therefore be analysed separately, rather than assuming that the ratio represents a single biological process.

The specific mechanistic hypothesis advanced here concerns PlGF: a gestational-age-inappropriate decline may constitute a delayed placental response to a sustained positive demand–supply imbalance. The corresponding mechanistic significance of sFlt-1 is not specified by the present framework; sFlt-1 remains a relevant, placentally derived biomarker whose interpretation is left open. Decomposition of the ratio is therefore motivated by the possibility that its components carry biologically distinct information, not by a claim to already know what each component represents.

Gestational age is also critical. PlGF normally rises and subsequently falls, whereas sFlt-1 increases towards term. A low absolute PlGF concentration late in pregnancy cannot therefore be interpreted using thresholds developed for suspected preterm pre-eclampsia. Gestational-age-adjusted multiples of the median and longitudinal trajectories are more appropriate for testing whether a fall is excessive relative to the physiological baseline.

### 4.2 A biphasic oxygen-sparing mechanism

We propose that a sustained positive fetal oxygen demand–supply imbalance elicits a biphasic response. Initially, the highly oxidative placenta may preserve its own oxygen consumption, leaving the fetus to experience much of the emerging deficit. If the mismatch persists, placental metabolic reprogramming towards glycolysis may reduce placental oxygen consumption and preserve a greater fraction of the limited oxygen supply for fetal transfer. Such adaptation does not necessarily resolve the underlying insufficiency; instead, it may redistribute part of its metabolic cost towards the placenta. The resulting hypoxic, energetic and endoplasmic-reticulum stress may contribute to reduced PlGF through HIF-2α- and integrated-stress-response pathways. We therefore propose declining PlGF as a delayed candidate signal of sustained demand–supply mismatch, rather than an immediate response to increasing fetal demand or a specific sensor of fetal hypoxia.

The first phase follows from the metabolic position of the placenta. The placenta is not a passive conduit: at term, it accounts for approximately 40% of oxygen uptake by the gravid uterus, with substantial expenditure on oxidative phosphorylation, protein synthesis and ion transport (Carter, 2000, 2015). In this phase, fetal compensation may precede a marked or clinically detectable angiogenic signal.

The second phase is based on placental metabolic plasticity. That placental metabolic reprogramming towards glycolysis can lower the organ’s own oxidative requirement and spare oxygen for fetal transfer was proposed explicitly by Illsley et al. (2010); the present model adopts that mechanism and places it within the demand–supply sequence rather than claiming it as new. The important claim here is not that metabolic reprogramming creates additional oxygen, nor that increased fetal transfer is guaranteed. Rather, reduced placental consumption may leave a greater fraction of a limited supply available to the fetus. Transfer still depends on maternal flow, diffusion, umbilical circulation and fetal oxygen-carrying capacity.

Metabolic reprogramming also does not necessarily restore placental oxygen sufficiency. It may instead redistribute the consequences of an unresolved supply limitation. A placenta relying more heavily on glycolysis may maintain part of its transfer function while accepting energetic inefficiency, endoplasmic-reticulum stress signalling and reduced capacity for its own biosynthetic work. In this sense, the adaptation may protect fetal oxygen availability temporarily while making the placental metabolic state increasingly abnormal.

One link in this sequence is not yet established and should be stated as an open question rather than assumed. If, in the first phase, trophoblast oxygenation is relatively preserved while the fetus bears the deficit, what signals the placenta to undertake oxygen-sparing reprogramming at all? Several candidate triggers can be named: a widening fetal–placental oxygen gradient sensed on the fetal side of the villous membrane; falling oxygenation specifically within the fetal capillary territory rather than in the trophoblast; reduced umbilical venous return or flow; or a fetal-derived metabolic or endocrine signal reporting the deficit back to the placenta. The framework does not depend on any particular one of these, but it does require that some signal couple the fetal deficit of the first phase to the placental adaptation of the second. Identifying that coupling—for example, by relating fetal-side and trophoblast oxygenation to the timing of metabolic and angiogenic change—is a specific experimental prediction, and a demonstration that placental reprogramming proceeds with no such fetal-to-placental signal would count against the biphasic mechanism (Section 8).

Experimental evidence provides plausible molecular links from this state to PlGF. Hypoxia can reduce PlGF expression and secretion in trophoblast models (Khaliq et al., 1999). During human trophoblast syncytialisation, reduced oxygen increased HIF-2α, and knockdown of HIF-2α—but not of HIF-1α—abrogated the hypoxia-associated reduction in PlGF expression (Fujii et al., 2017). This evidence supports HIF-2α involvement but does not demonstrate that it is the dominant mediator of falling circulating PlGF in the intact human placenta at term. In parallel, placental endoplasmic-reticulum stress activates the integrated stress response; ATF4 and ATF6β can negatively regulate PlGF transcription and secretion (Mizuuchi et al., 2016). PlGF may therefore integrate hypoxic, energetic and substrate-related stress rather than function as a specific oxygen sensor.

The same experimental work also reported increased placental PlGF expression in fetal growth restriction, which the authors interpreted as evidence of relative placental hyperoxia despite fetal compromise (Khaliq et al., 1999). This finding is neither confirmatory nor necessarily contradictory: it compares different biological levels and contexts—experimental hypoxia, tissue expression in fetal growth restriction, and, in the present argument, free maternal circulating PlGF—and it identifies a context-dependent observation that the proposed temporal model must explain prospectively rather than accommodate after the fact. Consistency with the model’s first phase (a fetal deficit coexisting with comparatively preserved trophoblast oxygenation and maintained tissue PlGF) is therefore a hypothesis to be tested, not a resolution to be asserted; it also underlines that placental tissue expression and free maternal circulating PlGF are not interchangeable, particularly where production, release and sFlt-1-mediated sequestration may differ. Critically, the phase must be classified independently—through metabolic and integrated stress-response signals and physiological oxygenation proxies—before circulating PlGF is interpreted, so that the biphasic reading cannot be fitted retrospectively to whichever direction PlGF happens to take.

We therefore state the prespecified, phase-specific signature in advance and keep cell and *ex vivo* evidence separate from clinical cohort association. The first required change is a fetal-side oxygen-support or adaptation signal—for example, a fall in umbilical venous oxygen delivery, or cerebroplacental redistribution—which precedes any circulating PlGF change; we specify temporal order without inventing a numerical lag. Operationally, a fetal-side oxygenation or haemodynamic change observed at a prespecified visit *t*, followed by a gestational-age-inappropriate PlGF change at the next prespecified visit *t* + 1, satisfies the proposed demand-first ordering; a simultaneous or earlier PlGF abnormality does not support that branch. Where the schedule uses the 32 + 0- and 36 + 0-week visits, the 4-week interval is an operational observation window, not an asserted biological lag. In the demand-predominant, initially uncomplicated sequence, placental PGF expression is expected to remain gestationally appropriate during the early fetal-side phase and to fall only in the later stress phase if the transcriptional arm operates (HIF-2α; ATF4/ATF6β; Fujii et al., 2017; Mizuuchi et al., 2016), producing a concordant fall in total and free circulating PlGF. An isolated fall in free PlGF with preserved total PlGF and preserved or increased tissue PGF would reject that transcriptional arm and instead favour sFlt-1 binding/sequestration, altered release or clearance—a materially different mechanism. The observation of Khaliq et al. (1999) of increased tissue PGF in established growth restriction is compatible with a competing tissue-level pattern but, without matched free and total circulating PlGF and temporal ordering, does not establish sequestration, altered release or clearance. It is therefore retained as a competing observation rather than fitted retrospectively to the proposed sequence. Early low total or tissue PGF, or an early uterine or placental abnormality preceding the fetal-side signal, would instead favour a pre-existing supply-side lesion. Total PlGF is treated as an imperfect localisation aid, not a pure assay of transcription.

The individual steps—placental oxygen consumption, metabolic plasticity, hypoxic and integrated stress-response downregulation of PlGF—are separately supported, but the complete sequence has not been demonstrated longitudinally in an initially uncomplicated human pregnancy at term. It is the central, falsifiable mechanistic hypothesis of this framework.

As a hypothesis rather than an established reading, declining PlGF is proposed to signal the metabolic cost incurred by a placenta preserving fetal oxygen availability under sustained mismatch, rather than defective early placentation alone—a decline that can, on this view, occur in a normotensive pregnancy with an appropriately grown fetus and no previously detectable uteroplacental abnormality.

### 4.3 PlGF as a candidate clinical readout

Observational evidence is compatible with this prediction. In a blinded prospective study of 378 apparently low-risk pregnancies assessed from 36 weeks, lower maternal PlGF was associated with intrapartum fetal compromise, abnormal fetal heart-rate patterns, abnormal cord gases, low 5-min Apgar scores and a composite adverse neonatal outcome (Bligh et al., 2016). A smaller study in high-risk term inductions similarly reported lower PlGF among pregnancies developing intrapartum compromise (Budak et al., 2018). In larger cohorts drawn from the same screening framework, a higher sFlt-1/PlGF ratio at 36 weeks was associated, in a graded fashion, with intrapartum fetal compromise in nominally low-risk pregnancies, an association that persisted after adjustment for pre-eclampsia (Farina et al., 2025); the maternal pre-eclampsia risk computed at 36 weeks was likewise associated with intrapartum fetal compromise and with spontaneous onset of labour, although that endpoint was not purely fetal (Cavoretto et al., 2025). These studies demonstrate association, not the proposed temporal mechanism, but they show that markers constructed to read maternal risk retain associations with fetal outcomes beyond their intended maternal-risk application, outside a population defined by maternal hypertension.

A systematic review of late-pregnancy PlGF found a consistent association with small-for-gestational-age birth and generally favourable, although heterogeneous, associations with caesarean delivery for fetal compromise, neonatal intensive-care admission and stillbirth (Sherrell et al., 2018). The heterogeneity is important. Low PlGF is neither a validated stand-alone measure of placental reserve nor a specific predictor of intrapartum hypoxia. Its value may lie in identifying a latent placental state when interpreted together with gestational age, fetal size, Doppler findings, maternal phenotype and, critically, its own trajectory.

Our hypothesis is therefore deliberately probabilistic: a gestational-age-inappropriate reduction in PlGF may index declining functional placental reserve, including when overt defective placentation, fetal growth restriction and maternal hypertension are absent. The trajectory may be more informative than a single value because the proposed signal is delayed and state-dependent. A rapid or excessive decline could identify transition from compensated proximity to the supply ceiling towards persistent positive imbalance.

### 4.4 Alternative and complementary mechanisms

Several explanations may produce the same circulating phenotype. Low circulating PlGF may reflect reduced transcription, impaired trophoblast differentiation, altered secretion, sequestration by sFlt-1, placental senescence, microscopic malperfusion or combinations of these processes. Oxygen regulation also varies by trophoblast model, gestational stage, exposure duration and whether tissue expression, total circulating PlGF or free PlGF is measured. These alternatives must be tested rather than absorbed into the oxygen-sparing hypothesis.

The anatomical geography of oxygenation may help explain why the demand-driven trajectory need not generate an sFlt-1 rise proportional to the fall in PlGF, but this remains a secondary prediction. A normal sFlt-1 concentration cannot be treated as proof that no placental lesion exists. Conversely, a high ratio cannot identify which component changed unless sFlt-1 and PlGF are reported separately.

Natural experiments such as twin-to-twin transfusion may generate useful hypotheses about unequal fetal demand, placental territories and angiogenic signalling, but they differ fundamentally from singleton physiology and should not be treated as direct proof. The decisive test requires longitudinal observation in initially normotensive, appropriately grown singleton pregnancies, with serial PlGF and sFlt-1, measures of placental oxygenation and metabolism, blinded placental histology and objective fetal endpoints.

## 5 Divergent maternal and fetal expressions

If placental sufficiency is a relational latent state, its clinical visibility depends on how each actor responds to it. The same positive imbalance can produce a maternal syndrome, a fetal consequence, both or—transiently—neither. This section develops why the maternal and fetal expressions are distinct, why they need not coincide and why the intensity of one is not a reliable measure of the other.

### 5.1 The maternal expression

The maternal syndrome is best understood as a host-dependent transformation of a placental signal rather than a direct readout of the imbalance itself. Excess sFlt-1 sequesters circulating VEGF and PlGF; the resulting reduction in free VEGF available to the maternal endothelium can produce hypertension, proteinuria and the systemic features of pre-eclampsia (Maynard et al., 2003). The signal is placental, but the phenotype is maternal.

Crucially, that phenotype depends not only on the magnitude of the signal but on the maternal endothelial and cardiovascular response to it. We denote this host-dependent effect-modifying function—the maternal response to angiogenic deprivation—as *V*_m_, a property of the maternal vertex rather than a third term added to the balance. Conceptually, the maternal phenotype can be represented as an interaction between the placental signal and a host-dependent response-modifying function, *V*_m_; the term is used as a conceptual effect modifier, not as a ‘gain’ implying a linear or directly measurable product. Blood pressure and the maternal cardiovascular phenotype (Thilaganathan and Kalafat, 2019), and ophthalmic artery Doppler indices (Sarno et al., 2021), may describe the resulting maternal state, but none directly measures the response slope itself. A given placental signal may therefore produce overt disease in one woman and remain clinically silent in another.

Two consequences follow. First, pre-eclampsia is a host-dependent maternal expression of placental signalling, not the necessary mediator of fetal compromise. Second, once established, the maternal syndrome may feed back onto supply: hypertension, endothelial dysfunction and the clinical management they prompt can further reduce effective uteroplacental perfusion, so that pre-eclampsia becomes a secondary amplifier of the very imbalance it signals. Mirror syndrome illustrates that a maternal phenotype can track a fetoplacental pathological process and may improve when that process is corrected, rather than directly reflecting the severity of fetal hypoxaemia (Braun et al., 2010).

A rival reading must be named here, where the host-response functions are first defined, rather than deferred: a single placental lesion of shared severity, filtered through variable maternal and fetal host responses, could reproduce the same divergence without any distinct latent balance. We do not treat this as a late caveat; the longitudinal mediation and multistate designs of Section 8.2 are presented below as the discriminating test that separates it from the proposed relational pathway.

### 5.2 The fetal response and the fetal consequence

The fetal side requires a distinction between the response and the consequence. The response is compensation: redistribution of cardiac output towards the brain, myocardium and adrenals; reduced activity and metabolic rate; and slowing of growth. These mechanisms either lower realised oxygen consumption (*C*_a_) or protect priority organs—without altering the underlying requirement (*C*_o_)—and their variability is a property of the fetal vertex. Because growth slowing is itself one such response, fetal growth restriction is better understood as an adaptation to, or a consequence of, impaired supply than as the defining feature of insufficiency. An appropriately grown fetus may simply have compensated by other means or may have approached the supply ceiling only recently.

The consequence—compromise—emerges when compensation is exhausted: progressive hypoxaemia, metabolic acidaemia, neonatal encephalopathy and, at the extreme, stillbirth or intrapartum death. Fetal compromise is therefore not the fetal response but the failure of that response. The sequence is orderly: sustained positive imbalance, then fetal compensation, then exhaustion of reserve, then compromise. A framework that reads only the endpoint of this sequence, or only its maternal counterpart, cannot distinguish a compensating fetus from a failing one.

### 5.3 Conditional divergence

Both maternal and fetal expressions arise from the same underlying placental state, but they are modified by different host-specific response systems. Conditional on the measurable features of placental dysfunction, the severity of one phenotype may therefore remain only incompletely coupled to the severity of the other. Maternal disease can feed back on placental supply, but neither maternal silence nor maternal severity is sufficient, by itself, to infer the fetal state. A strong maternal response does not necessarily imply fetal protection or fetal compromise, just as maternal silence does not establish fetal safety.

These response systems can be named, but the notation must be disciplined so that it does not become an all-purpose explanation. We write the maternal response-modifying function for placental signalling as *V*_m_ and the fetal response-modifying function—the fetus-specific response to a given magnitude and duration of positive imbalance—as *V*_f_, and treat them as conceptual effect-modifying functions rather than ‘gains’, not as free latent parameters to be invoked after the fact but as placeholders for *prespecified, measurable* modifiers. On the maternal side, these include the cardiovascular and endothelial indices already used to describe pre-eclampsia risk; on the fetal side, measures of compensatory reserve such as cerebroplacental ratio and the fetal cardiovascular response. A model that lets *V*_m_ and *V*_f_ absorb any pattern of concordance or divergence is untestable; the framework therefore commits to defining these modifiers in advance and to requiring that any divergence they explain replicate in an external cohort rather than being fitted within one. It must also be conceded that a rival account—a single placental lesion of shared severity, filtered through variable maternal and fetal host responses—can be made observationally almost equivalent, because it too invokes host modifiers. The novelty of the present model cannot therefore rest on *V*_m_ and *V*_f_; it rests on the three-level separation and the specific, prespecified way the divergence is to be tested (Section 8.2).

This incomplete coupling has an immediate clinical corollary: the intensity of the maternal expression is not a reliable measure of fetal jeopardy. Severe pre-eclampsia and severe fetal compromise should not be treated as interchangeable indicators of how endangered a pregnancy is.

Maternal silence, moreover, has two distinct sources, and separating them sharpens the argument. The first is host silence: a low *V*_m_ converts even a substantial signal into little or no maternal phenotype. The second is temporal: the maternal and fetal clocks differ. Maternal clinical expression often evolves over days to weeks, whereas fetal decompensation under acute or repetitive oxygen limitation can occur over minutes to hours, particularly during labour. The biphasic, delayed-signal mechanism proposed in Section 4.2 reinforces this second silence: because the circulating signal may lag the imbalance that generates it, a fetal deficit can precede any strong maternal readout.

Read through this lens, the familiar clinical dissociations become more intelligible. Some pregnancies show fetal growth restriction without pre-eclampsia; others show pre-eclampsia with a normally grown fetus; others show both. These configurations need not represent wholly separate disease processes; in at least a subset of pregnancies, they may represent different projections of a shared placental imbalance onto differently calibrated maternal and fetal response systems (**Figure 3**).

**Figure 3:**
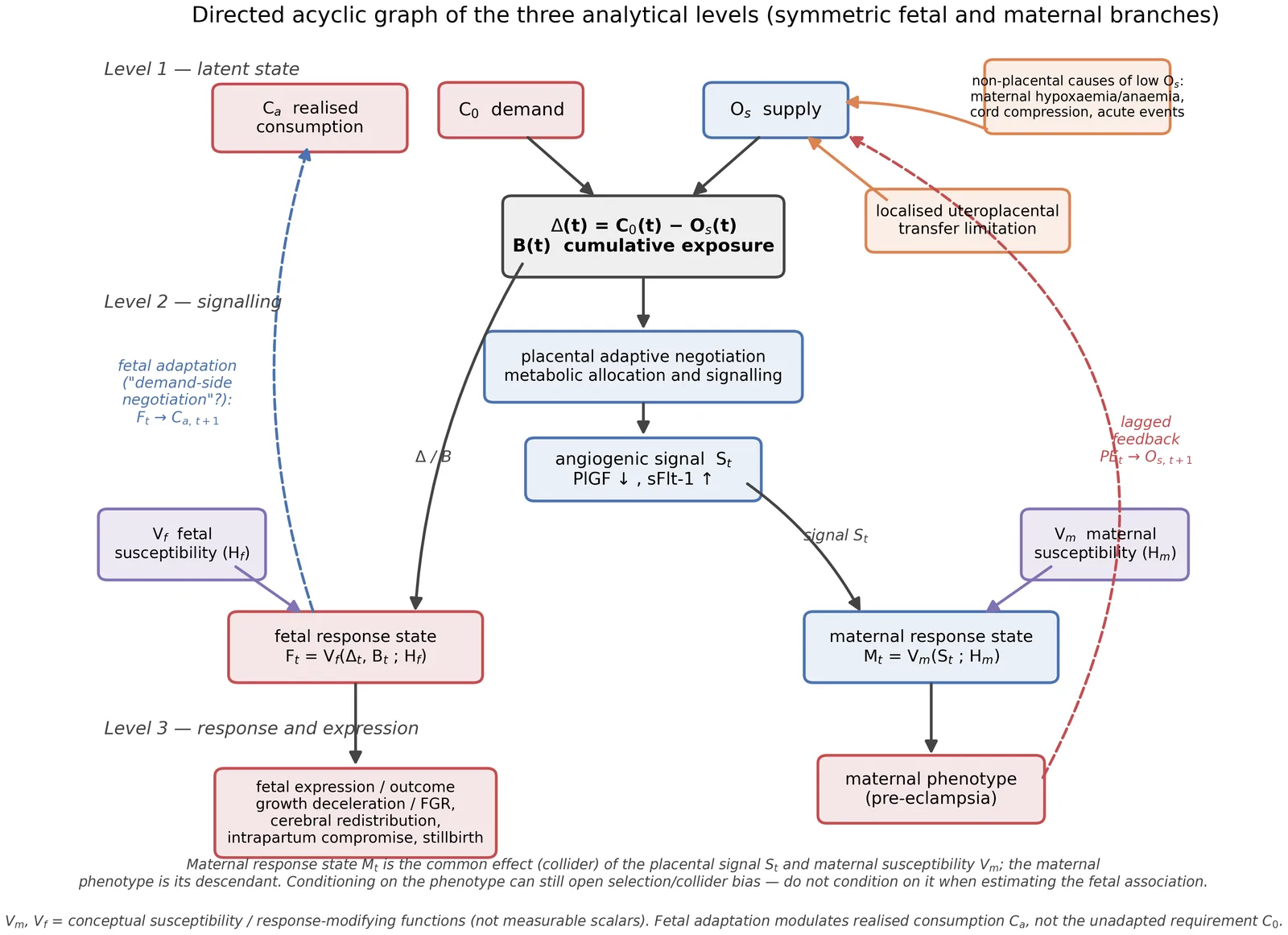
Directed acyclic graph of the three analytical levels, drawn with symmetric fetal and maternal branches. *Level 1 (latent state):* the unadapted fetal oxygen requirement *C*_o_(*t*) and the system-level supply capacity *O*_s_(*t*) define the instantaneous imbalance Δ(*t*) = *C*_o_(*t*) − *O*_s_(*t*) and its cumulative exposure *B*(*t*); the fetus’s realised oxygen consumption *C*_a_(*t*) is shown separately, because fetal adaptation lowers realised consumption without altering the unadapted requirement. Non-placental determinants of low *O*_s_—maternal hypoxaemia or anaemia, cord compression, acute maternal hypoxaemic events—are shown as inputs distinct from a uteroplacental limitation, so that localisation is an explicit step. A localised uteroplacental transfer limitation is shown as one such determinant of low *O*_s_, distinct from the non-placental determinants, so that localisation is an explicit step. *Level 2 (signalling):* sustained imbalance drives placental adaptive negotiation, of which the angiogenic signal *S*_t_ (PlGF ↓, sFlt-1 ↑) is the maternal-facing output. ‘Negotiation’ is used as functional shorthand for adaptive changes in placental metabolism, transport, resource allocation and signalling; it implies neither agency nor intentionality nor a single established molecular programme, of which metabolic reprogramming (Section 4.2; **Tables 4**, **5**) is one possible mechanism. *Level 3 (response and expression):* the two sides are symmetric. On the maternal side (right), the placental signal *S*_t_ and a maternal susceptibility function *V*_m_ combine into a maternal response state *M*_t_ = *V*_m_(*S*_t_; *H*_m_), of which the maternal phenotype (pre-eclampsia) is the downstream expression. On the fetal side (left), the effective deficit—Δ(*t*) and its cumulative exposure *B*(*t*), not the PlGF proxy—and a fetal susceptibility function *V*_f_ combine into a fetal response state *F*_t_ = *V*_f_(Δ_t_, *B*_t_; *H*_f_), of which the fetal expression is the downstream outcome: growth deceleration or fetal growth restriction, cerebral redistribution, intrapartum compromise and stillbirth, which may occur even in an appropriately grown fetus. *V*_m_ and *V*_f_ are conceptual susceptibility/response-modifying functions that modulate the response to the input, not measurable scalars and not separate causes. The maternal response state *M*_t_ is the common effect (collider) of the placental signal *S*_t_ and maternal susceptibility *V*_m_, and the maternal phenotype is its descendant; conditioning on the phenotype (for example, by adjusting for pre-eclampsia) can still open selection/collider bias, so it is not conditioned upon when the fetal association is estimated; its analytic role is otherwise time- and question-specific (Section 8). The graph is acyclic because both feedback paths are time-indexed and drawn as lagged edges: the maternal phenotype at time *t* lowers *O*_s_ at *t* + 1 (right), and the fetal response state *F*_t_ modulates the realised consumption *C*_a_ at *t* + 1 (left)—fetal adaptation acting on realised consumption, not on the unadapted requirement *C*_o_. It is the object from which the mediation and multistate analyses of Section 8.2 are specified.

## 6 Redefining the reference endpoint of placental oxygen-transfer function

If placental function is relational, and if its maternal and fetal expressions can diverge, then the endpoint by which that function is judged requires re-examination. Three clinical questions are routinely conflated, yet each requires a different endpoint (**Table 2**).

**Table 2:** Three clinical questions in gestational disease and their appropriate endpoints.

Clinical question	Appropriate endpoint
Is the mother affected?	Pre-eclampsia, HELLP, maternal organ dysfunction
Is fetal support becoming inadequate?	Compensatory redistribution, altered growth trajectory, metabolic acidaemia, encephalopathy, death
Is the placenta performing its transfer function?	Adequacy of oxygen transfer relative to current fetal demand

Clinical screening frameworks have often operationalised placental dysfunction primarily through the maternal hypertensive phenotype, although fetal growth, Doppler and biochemical markers increasingly complement that endpoint. The limitation arises when absence of the maternal phenotype is interpreted as evidence of adequate fetal support. This is not a failure of calibration; on its own target, the maternal model performs well. It is a limitation of the chosen endpoint: a latent state with more than one expression is being read through only one of them.

We therefore propose that the reference endpoint for placental oxygen-transfer function should shift from the presence of a maternal syndrome to the adequacy of fetal support. This does not subordinate maternal outcomes. Maternal and fetal morbidity remain co-equal clinical outcomes, each important in its own right. What changes is the criterion by which placental oxygen-transfer function itself is judged: the question “Is the placenta doing its job?” is answered in terms of fetal adequacy, because the transfer function exists to meet fetal requirement. This reference endpoint is *normative*—the criterion by which transfer function is defined—and must be distinguished from the *measurable validation* endpoints against which the model is tested, namely, the adjudicated, least management-dependent fetal outcomes below, none of which is wholly specific to placental oxygen transfer. The two roles should not be conflated.

Testing this reframing requires endpoints graded by how far they can be separated from clinical management and interpreted with the recognition that none is wholly specific to placental oxygen transfer. The primary endpoints should be the least management-dependent: metabolic acidaemia at birth, defined by a prespecified consensus threshold—umbilical arterial pH below 7.00 with a base deficit in the extracellular fluid compartment (BDecf) of at least 12 mmol/L on a validated paired cord sample (Ayres-de-Campos et al., 2015); neonatal encephalopathy; and antepartum or intrapartum death. These differ in frequency, causal specificity and mode of ascertainment, and are defined and analysed separately rather than placed on a single implied severity scale. Because encephalopathy and death in particular can also arise from acute extraplacental events—cord accidents, placental abruption, intrapartum infection, feto-maternal haemorrhage—these events must be adjudicated and, where identifiable, excluded or analysed separately, so that the endpoint reflects failure of oxygen transfer rather than a distinct acute insult. Cord-gas sampling should be standardised, and the components of any composite reported and analysed individually as well as together, because a composite can be driven by its least placenta-specific element. Intermediate endpoints—Doppler indices, deviation from an individualised growth trajectory and cardiotocographic abnormality—are informative but less specific. Operative delivery for suspected fetal compromise is a frankly management-dependent proxy: it depends on cardiotocographic interpretation and local practice and can be both produced and prevented by clinical behaviour. A framework whose central claim concerns fetal adequacy must therefore be tested primarily against the least management-dependent endpoints, reserving the management-dependent ones for context.

A corollary concerns interventions that target the signal rather than the imbalance. Targeted reduction of circulating sFlt-1 has been associated with improvement in maternal disease markers and pregnancy prolongation in a pilot trial (Thadhani et al., 2026). However, normalisation of angiogenic signalling does not, by itself, demonstrate restoration of placental transfer capacity or correction of the underlying demand–supply imbalance. Maternal-phenotype-based surveillance could become less informative if biochemical improvement is not accompanied by restoration of placental transfer capacity. Trials of such interventions should therefore report objective fetal endpoints, not maternal improvement and pregnancy prolongation alone.

## 7 Relationship to existing frameworks

The relational model is intended to extend and connect existing accounts of gestational disease rather than to replace them. Several of its physiological components are not themselves new, and it is important to say so plainly. The placenta has long been described as matching fetal requirement to maternal supply through placental nutrient sensing (Díaz et al., 2014); the capacity–load model of Desoye and Wells (2021) frames the placenta as buffering a largely maternal metabolic load against its own homeostatic capacity, adapting to fetal signals such as insulin that can themselves raise fetal oxygen demand; Eskild et al. (2016) proposed that the fetal oxygen requirement rises towards term, that limitation of maternal supply can generate fetoplacental hypoxia even with a normal-sized placenta and that pre-eclampsia and its angiogenic factors may be a response to that hypoxia rather than its cause; oxygen-sparing metabolic reprogramming was proposed by Illsley et al. (2010); labour has been framed as a demand–supply stress test that can unmask a relative uteroplacental insufficiency (Turner et al., 2020; Ghi et al., 2024); and a fetal-centred reading of pre-eclampsia was advanced by Espinoza and Espinoza (2011). The contribution claimed here is therefore not the introduction of demand–supply physiology—close antecedents exist—but a specific combination: the generalisation of a capacity–load logic to oxygen-transfer sufficiency across gestation and labour; its integration into a model with three explicitly separated levels (a latent functional state, placental signalling, and partly decoupled maternal and fetal expressions); the separation of the causal trajectory from the clinical expression; the two sources of maternal silence, host-dependent and temporal; the biphasic delayed-PlGF sequence; and fetal adequacy as the reference endpoint. No single one of these is the novelty; the combination is.

The two-stage model of pre-eclampsia describes poor early placentation giving rise to a maternal syndrome through placental stress and antiangiogenic signalling (Roberts and Hubel, 2009). It accounts powerfully for early-onset, placentally driven disease. What it does not specify is the fetal axis or the demand side of the relation: it explains why a poorly built placenta signals, but not why an apparently well-built placenta can become insufficient as fetal requirement rises, nor why fetal compromise can occur without the maternal syndrome. The demand–supply formulation supplies the missing term—fetal requirement—and makes the fetal consequence an explicit endpoint rather than a downstream annotation.

The ISSHP framework recognises angiogenic imbalance as a marker of uteroplacental dysfunction but retains it within a diagnosis anchored on maternal hypertension (Magee et al., 2022). This is the precise point at which the present model diverges: if the imbalance is real and the maternal host response is low, the same uteroplacental dysfunction may exist and may threaten the fetus, without crossing the hypertensive threshold that would bring it to clinical attention. We treat the angiogenic signal as an index of a latent state rather than as a feature of a maternal diagnosis.

The competing-risks model has provided the most influential screening architecture of the past decade, calibrating the timing of pre-eclampsia against gestational age with considerable accuracy (Wright et al., 2020). Its endpoint, however, is singular and maternal. Read through the present lens, the same machinery that arbitrates between delivery and the onset of maternal disease could be extended to represent more than one outcome of a single latent imbalance. Two axes must be kept separate here, because conflating them weakens the argument: the causal trajectory by which the imbalance arises (supply-driven, demand-driven or mixed) and the expression through which it becomes clinically visible (a maternal syndrome, fetal compromise, both or neither). The trajectory concerns aetiology; the expression concerns which actor manifests the shared state. The shift the model proposes is thus from competing risks in time, resolved on a single maternal endpoint, to competing expressions across actors of one latent state. That the model’s own readout already predicts fetal compromise, including in nominally low-risk pregnancies (Farina et al., 2025; Cavoretto et al., 2025), and that a competing-risks formulation extends to placental-dysfunction-related stillbirth (Nicolaides et al., 2022), suggests that the fetal expression is already partly visible within a maternally calibrated instrument.

Two accounts are closest. The capacity–load model of Desoye and Wells (2021) shares the central intuition—an active placenta mediating between what the pregnancy demands and what can be supplied—but was developed for a largely maternal-metabolic substrate load rather than the fetal oxygen requirement placed at the centre here; the present framework generalises that logic to oxygen-transfer sufficiency, separates the latent balance from signalling and expression, makes fetal adequacy the reference endpoint and adds the biphasic delayed PlGF mechanism. The study of Eskild et al. (2016) is the closest account on the demand side and on reading pre-eclampsia as a response rather than a cause, and its proximity should be stated generously: they argued that fetal oxygen requirement rises towards term, that a sub-optimal maternal supply can generate feto-placental hypoxia even with a normal-sized placenta and that the angiogenic factors may be responses to that hypoxia—three of this framework’s premises were present already in 2016. The present model differs in mechanism and formalism rather than orientation: Eskild’s proposed adaptive output is structural and angiogenic expansion, whereas ours is metabolic oxygen-sparing with a directionally distinct, delayed PlGF decline; beyond this, we add a formal relation on a common oxygen scale, the three-level separation, fetal adequacy as endpoint and an explicit falsification programme (**Table 3**).

**Table 3:** Claim-by-claim comparison of the present framework with the closest existing accounts.

A Construct terms.			
Framework	Unit of insufficiency	Demand term	Supply term
Present (relational *C*_o_/*O*_s_)	Oxygen-transfer relation (system → localised uteroplacental)	Fetal O_2_ requirement *C*_o_ (counterfactual)	System-level capacity *O*_s_
Eskild et al. (2016)	feto-placental hypoxia	Fetal O_2_ demand rising to term	Maternal supply
Desoye and Wells (2021)	Capacity–load (substrate)	Metabolic load, largely maternal	Placental homeostatic capacity
Illsley et al. (2010)	Metabolic reprogramming	–	–
Espinoza and Espinoza (2011)	PE as fetal adaptive response	Fetal demand	–
Maternal cardiovascular (Thilaganathan and Kalafat, 2019)	Maternal cardiovascular capacity	Demands of pregnancy on maternal circulation	Maternal cardiac reserve
Maturation/PMIP (Chueh and Chang, 2026)	Mature placenta overwhelmed	Largely maternal demand	Maturational ceiling
Shared-lesion rival	Single placental lesion of shared severity	–	–

B Causal mechanism and maternal–fetal relation.			
Framework	Causal mechanism	Maternal–fetal relation	
Present (relational *C*_o_/*O*_s_)	Biphasic oxygen-sparing → delayed transcriptional PlGF fall	Decoupled via *V*_m_/*V*_f_ and a temporal lag	
Eskild et al. (2016)	Placental enlargement + angiogenesis; PE as response	PE = maternal response	
Desoye and Wells (2021)	Buffering of load; fetal-signal adaptation	Developed for diabetes/obesity	
Illsley et al. (2010)	Glycolytic oxygen-sparing	–	
Espinoza and Espinoza (2011)	Fetal-to-maternal signalling (adenosine, antiangiogenic)	Correct direction; PE = maternal manifestation	
Maternal cardiovascular (Thilaganathan and Kalafat, 2019)	Maternal haemodynamic limitation	Maternal phenotype primary	
Maturation/PMIP (Chueh and Chang, 2026)	Third-trimester maturational inflection	Maternal phenotype = outcome	
Shared-lesion rival	One lesion → both phenotypes via host responses	Direct maternal ↔ fetal association pre-onset	

C Measurable prediction and distinguishing observation.			
Framework	Measurable prediction	Distinguishing/falsifying observation	
Present (relational *C*_o_/*O*_s_)	Free-PlGF trajectory (with total PlGF, sFlt-1) → adjudicated fetal endpoint	Fetal-side signal precedes a concordant total+free PlGF fall in an initially normal singleton	
Eskild et al. (2016)	Placental enlargement, angiogenic change	Predicts enlargement/angiogenesis, opposite to the present oxygen-sparing signature	
Desoye and Wells (2021)	Placental response to insulin/substrate	Load is maternal-metabolic, not fetal O_2_; no fetal-first PlGF ordering	
Illsley et al. (2010)	Substrate-mix shift	Adopted component, not a rival	
Espinoza and Espinoza (2011)	Fetal-derived maternal signals	Close antecedent; lacks the demand–supply balance and biphasic PlGF route	
Maternal cardiovascular (Thilaganathan and Kalafat, 2019)	Maternal cardiovascular indices	Locates origin in the maternal circulation, not the transfer ↔ demand relation	
Maturation/PMIP (Chueh and Chang, 2026)	Maturation inflection	No fetal-signal-first ordering; demand framed as maternal	
Shared-lesion rival	Direct, non-*O*_s_-mediated association	Predicts a direct maternal contribution before maternal onset (Section 8.2)	

The maternal cardiovascular model (Thilaganathan and Kalafat, 2019) locates the late maternal phenotype in the maternal circulation and describes the maternal response function with a precision we do not attempt; it is complementary rather than alternative—it characterises one vertex in detail, while the present relation names the balance that determines the fetal consequence—and is incorporated rather than rejected.

Two recent frameworks require specific differentiation. Espinoza and Espinoza (2011) proposed pre-eclampsia as a maternal manifestation of a fetal adaptive response and already invoked fetal-derived signalling (adenosine, antiangiogenic factors); it is a close antecedent that lacks the demand–supply balance and the biphasic, oxygen-sparing route the present framework adds. The placental-maturation inflection-point model (Chueh and Chang, 2026) agrees that late disease can arise without a new structural lesion, but frames the exceeding demand as chiefly maternal, whereas our demand term is fetal and predominantly for oxygen and we separate the latent imbalance from the maternal expression and the fetal consequence; a parallel synthesis (Bilginer and Çetin, 2026) re-examines placental origins but not a relational definition of function. The distinguishing move of the present framework is to define placental function itself and to make fetal adequacy its reference endpoint.

To make the incremental claim checkable rather than a narrative assertion of ‘combination’, **Table 3** compares the present framework with its closest antecedents across seven dimensions.

After auditing the closest antecedents, we do not claim that rising fetal demand, fetal-to-placental signalling, oxygen-sparing metabolism or a late PlGF decline is individually unique; each has a clear precedent. The distinctive prediction is *conjunctive* and is not prespecified by the cited alternatives: in an initially normotensive, appropriately grown singleton with a normal early trajectory and no early evidence of a major supply-side lesion, a prespecified fetal-side oxygen-support or adaptation signal should precede a gestational-age-inappropriate, concordant fall in total and free maternal PlGF. This pattern is compatible with reduced PGF production rather than isolated sFlt-1 sequestration. If the temporal ordering and assay pattern do not replicate, or prove equally expected under a simpler fixed-lesion or maturation account, the framework should be treated as an integrative heuristic and research programme rather than as a distinct mechanistic theory.

## 8 Testable predictions and falsification

A framework of this kind earns its place only if it can be shown to be wrong. The predictions below are grouped by what they test—the biomarkers, the clinical expressions and the mechanism itself—and are followed by the conditions under which each level of the hypothesis would fail.

### 8.1 Measurement, identification and the primary estimand

To forestall the analytic flexibility a latent-variable proposal invites—a threshold, a reserve function, a cumulative term, transformations of the proxies and the choice of time windows all selected after the data are seen—we prespecify a single primary estimand and separate it sharply from the mechanistic claim.

#### 8.1.1 Primary (prognostic) estimand

At a fixed 36 + 0-week landmark (a landmark design; Putter and van Houwelingen, 2022), among ongoing singleton pregnancies that were initially normotensive, have no major fetal anomaly and remain appropriately grown, the predictor is a gestational-age-standardised PlGF signature measured entirely before or at time zero: free-PlGF multiples of the median at the landmark and their prespecified fixed-window pre-landmark slope, interpreted jointly with total PlGF and sFlt-1. The primary outcome is an adjudicated fetal-compromise composite through birth and the first seven neonatal days—umbilical-artery metabolic acidaemia (pH < 7.00 with BDecf ≥ 12 mmol/L), moderate or severe neonatal encephalopathy, or fetal or neonatal death—whose components are also reported separately. The estimand is the absolute risk of that outcome under observed care as a prespecified function of the PlGF signature, evaluated by calibration and discrimination and confirmed by external validation in an independent cohort rather than a random split of one. No value arising after time zero enters the primary predictor; in particular, a slope to delivery is not used, because delivery is management-dependent and would import post-time-zero information. Better prediction would support the clinical usefulness of the signature; it would not identify *C*_o_, *O*_s_, Δ, the reserve *R* or uteroplacental localisation.

#### 8.1.2 Prespecified analysis

Serial measurements are taken at 32 + 0 and 36 + 0 weeks, and the landmark is 36 + 0. The predictors are log_2_ free-PlGF MoM at 36 weeks, the slope in log_2_ free-PlGF MoM between 32 and 36 weeks, and log_2_ total-PlGF MoM and log_2_ sFlt-1 MoM at the landmark. The primary model is logistic with linear main effects only—no thresholds, splines or interactions selected after inspecting the data. Missing data are handled by multiple imputation under a missing-at-random assumption, with complete-case and pattern-mixture (MNAR) analyses as prespecified sensitivity checks. Discrimination and calibration are reported as the area under the ROC curve, the Brier score, and the calibration slope and intercept, in a genuinely external validation cohort rather than a random split of one. This single specification is fixed in advance; it is not a menu from which a best-fitting form is later chosen. Full estimation and missing-data algorithms are locked in the prospective analysis plan before data access and are not presented here as validated features of the biological model.

#### 8.1.3 Measurement model

Each latent construct is assigned observable component indicators, their timing, an analytic role, the expected direction of measurement error and what they cannot establish (**Table 1**). Umbilical venous oxygen delivery, fetal oxygen extraction from Doppler/Fick estimation and MRI oximetry quantify different realised components of oxygen transport; ultrasound flow is sensitive to vessel-diameter and timing error, Fick uptake requires paired arterial–venous contents under near-steady-state conditions and is usually obtained peri-delivery (Cetin et al., 2020), and MRI oximetry depends on calibration, haematocrit/HbF, motion and model assumptions (Rodríguez-Soto et al., 2018; Saini et al., 2020; cf. Schabel et al., 2022)—so none alone identifies integrated capacity *O*_s_, the unadapted requirement *C*_o_ or reserve *R*. The analytic role of the maternal phenotype is time- and question-specific—an eligibility criterion at the landmark, and after it a possible mediator, management trigger or time-varying confounder—and is therefore not treated as a universal collider. Fetal activity is not used as an indicator of *C*_o_, since it may already be an adaptive response.

#### 8.1.4 Separating prognosis, mechanism and causality

The landmark analysis above is prognostic. The mechanistic hypothesis (Section 4.2) requires the time-ordered, localisation-specific predictions set out there and is not inferred from predictive performance. No intervention-effect estimand is advanced in this Hypothesis and Theory article; target-trial emulation (Hernán et al., 2025) is identified as the appropriate design for a *future* causal comparison of explicitly defined monitoring-and-delivery policies, at which point eligibility, assignment, follow-up, time-updated management, competing events (elective delivery for antenatal indications, intrauterine death, acute extraplacental catastrophes) and informative selection would have to be fully specified. The latent imbalance is not itself a well-defined intervention, and association is not presented as evidence of mechanism or causality.

#### 8.1.5 Relative maternal silence, defined operationally

Relative maternal silence is defined operationally, not mechanistically: at the 36 + 0-week landmark, no maternal pre-eclampsia or hypertensive syndrome has been diagnosed under observed care. It is the observed absence of maternal expression—not an inferred normal *V*_m_, a normal angiogenic profile, or evidence of fetal safety—and is framed as a hypothesis about residual fetal risk after the recommended multimodal clinical and ultrasound assessment, not a screening recommendation or a substitute for fetal surveillance. We note the AFFIRM experience as a caution that intensifying one surveillance pathway can alter intervention rates without a demonstrated reduction in stillbirth (Norman et al., 2018).

### 8.2 Predictions concerning biomarkers

First, in normotensive pregnancies with an appropriately grown fetus, a gestational-age-inappropriate reduction in PlGF, or an accelerated downward trajectory, will anticipate objective fetal endpoints—cord metabolic acidaemia, neonatal encephalopathy and intrapartum or antepartum death—more strongly than a single absolute value; a low 5-min Apgar score may serve as a supportive but less specific indicator, given its many extraplacental determinants. Second, the PlGF trajectory will be more informative than a single measurement, consistent with a delayed, state-dependent signal. Third, decomposition of the sFlt-1/PlGF ratio should reveal route- and endpoint-dependent contributions of its components. The absolute predictive performance of the ratio may exceed that of either component in some populations; the distinctive prediction is therefore not universal superiority of an individual component, but differential temporal and outcome associations of sFlt-1 and PlGF. The specific prediction is that a gestational-age-inappropriate PlGF decline will track objective fetal endpoints associated with a sustained positive demand–supply imbalance. Associations of sFlt-1 with maternal or fetal endpoints should be analysed separately, without assigning it a predefined mechanistic role.

### 8.3 Predictions concerning clinical expressions

Fourth, maternal host characteristics will modify the association between the placental signal and the maternal phenotype more than they modify the association between the underlying imbalance and fetal compromise. Fifth, the prediction concerns a causal pathway, not mere incremental prediction—a distinction the model must draw itself, because it permits maternal severity to carry genuine information about the fetus. Once established, pre-eclampsia can lower *O*_s_, through maternal haemodynamic change and the clinical management it prompts, and thereby raise Δ; a bare finding that maternal severity predicts the fetal outcome is therefore consistent with the relational model and does not discriminate between it and its rivals. The discriminating test is longitudinal and mediational. Before the onset of overt maternal expression, maternal host response should add little to prediction of the objective fetal outcome once shared measures of placental state are accounted for. After onset, whatever predictive contribution maternal severity carries should be largely mediated by the fall in *O*_s_ it induces, rather than acting as a direct marker of a shared lesion of fixed severity. The principal rival reading—that pre-eclampsia and fetal compromise are two faces of one placental lesion of common severity—instead predicts a substantial direct, non-*O*_s_-mediated association between maternal severity and fetal compromise, present before as well as after maternal onset. Distinguishing these requires mediation or multistate models with time-updated covariates, not a single incremental-value statistic; because *C*_o_ and *O*_s_ are measured imperfectly, the discriminating quantity is the size and timing of the direct maternal contribution, not its absence. Because the maternal and fetal host modifiers standing in for *V*_m_ and *V*_f_ are prespecified and measurable (Section 5.3), any divergence attributed to them must replicate in an external cohort to count as support rather than *post hoc* accommodation; and it must be acknowledged that, where the rival shared-lesion account fits the same data through its own host terms, the two models may be only weakly separable and should be reported as such. This is preliminarily compatible with existing data rather than established by them: the 36-week maternal risk has been reported to predict intrapartum fetal compromise most weakly, where maternal expression is strongest and more strongly where it is muted, though these analyses are observational and conditioned on the indication for induction (Farina et al., 2025; Cavoretto et al., 2025).

### 8.4 What would falsify the framework and at which level

Because the framework makes three nested claims, a negative result must be assigned to the correct level; failure of one does not necessarily falsify the others (**Table 4**). Δ = *C*_o_ − *O*_s_ defines the latent physiological balance; it is not itself a statistically identified estimand. The falsifiable empirical claims concern the prognostic usefulness of the prespecified observable signature, the proposed temporal mechanism and the PlGF readout.

**Table 4:** The three nested claims of the framework and what would falsify each.

Level	What would falsify it
Primary prognostic proxy claim	The prespecified landmark PlGF signature model fails external validation and provides no useful improvement over prespecified clinical and supply-proxy comparators.
Biphasic mechanism	Sustained imbalance does not produce placental metabolic reprogramming or oxygen-sparing; or the proposed acute-then-chronic sequence, including a fetal-to-placental trigger coupling the two phases, cannot be demonstrated.
PlGF hypothesis	PlGF does not track the metabolic transition, or does not associate independently with the fetal endpoints.

**Table 5:** Summarises the epistemic status of the framework’s principal claims.

Claim	Status	Key evidence
Placenta consumes ~40% of uterine oxygen uptake	Established (physiological)	Carter (2000, 2015)
Placental metabolism can reprogramme towards glycolysis	Experimental support	Illsley et al. (2010)
Hypoxia and endoplasmic-reticulum stress reduce PlGF (HIF-2α; ATF4/ATF6β)	Demonstrated in experimental systems	Khaliq et al. (1999); Fujii et al. (2017); Mizuuchi et al. (2016)
In the cited placental-expression study, the raised ratio was sFlt-1-driven in pre-eclampsia and PlGF-driven in fetal growth restriction	Observational, context-dependent	Gaccioli et al. (2023)
Low PlGF associates with fetal compromise in low-risk term pregnancies	Observed in prospective cohorts; heterogeneous associative evidence	Bligh et al. (2016); Sherrell et al. (2018); Farina et al. (2025)
Oxygen-sparing reprogramming preserves fetal oxygen availability and subsequently lowers PlGF	Integrative hypothesis	Present work
Placental oxygen-transfer function is a demand–supply relation, not a fixed property of the organ	Integrative inference	Present work
Pre-eclampsia is a host-dependent expression; fetal compromise is a distinct projection of the same state	Integrative inference	Present work; cf. Espinoza and Espinoza (2011)
A biphasic sequence lowers PlGF without a new lesion; declining PlGF is a delayed signal of sustained *C*_o_ > *O*_s_	Proposed here; untested	Present work
The reference endpoint of placental oxygen-transfer function should be fetal adequacy	Normative proposal	Present work

Epistemic status of the framework’s principal claims.

Such a result would reject the proposed proxy-based detection strategy; it would not demonstrate that a fetal demand–supply imbalance cannot exist. The first row deserves a statistical caution, because the prognostic claim is easy to test in the wrong form. Δ is a *difference*, not a multiplicative interaction; the absence of a *C*_o_ × *O*_s_ interaction term would not falsify it. Nor can the relational content be captured by adding a linear term *C*_o_ − *O*_s_ to a model that already contains the main effects of *C*_o_ and O_s_, since that term is a linear combination of the same predictors and adds no information. The primary test is therefore the single prespecified landmark model of Section 8, judged against a supply-only model and an unconstrained additive model in the *C*_o_ and *O*_s_ proxies and the maternal phenotype, by out-of-sample performance in external validation rather than in-sample fit. *B* and *R* remain conceptual constructs of the model, not a menu of competing statistics to be selected after the data are seen; latent-balance formulations (a cumulative-exposure or fractional-reserve statistic, or an explicit latent model) are reserved for secondary, separately registered analyses and are not part of the primary test. A null result at the primary level constrains the balance formulation, not the individual physiology.

### 8.5 The decisive longitudinal test and its controls

The central sequence—an acute fetal-predominant deficit, followed by placental oxygen-sparing adaptation, followed by a delayed fall in circulating PlGF—has not been demonstrated longitudinally in an initially uncomplicated human pregnancy. Demonstrating it requires prospective measurement and prespecified adjustment, stratification and sensitivity analyses addressing the alternative explanations for a late PlGF fall: pre-existing defective placentation, occult microscopic malperfusion, placental senescence, the physiological decline of PlGF towards term, altered PlGF production as distinct from release and sequestration by sFlt-1.

This should not be presented as a single study. The framework is falsifiable at three levels of maturity. Proxy-level predictions are testable now—that a gestational-age-inappropriate PlGF decline and its trajectory precede the least management-dependent fetal endpoints of Section 6, and that the prespecified landmark model of Section 8 outperforms supply alone. The compartmental mechanism—that reprogramming spares fetal oxygen while trophoblast oxygenation is comparatively preserved—is testable *ex vivo* but not yet *in vivo*: no technique separates fetal-capillary from trophoblast oxygenation in the intact human placenta, and quantitative placental T2* mapping yields only a whole-placenta oxygenation signal (Schabel et al., 2022). A defensible near-term design is therefore a longitudinal cohort of initially normotensive, appropriately grown singletons with serial PlGF and sFlt-1, global placental oxygenation and metabolic imaging, and histology read blind to the angiogenic result—testing the realistic chain (a fetal oxygen deficit, a fetal-derived signal, a trophoblastic metabolic and integrated-stress-response shift, a delayed PlGF fall) rather than a compartment separation the methods cannot yet deliver.

### 8.6 Methodological safeguards

Four safeguards separate a genuine test from an artefact. First, biomarkers must be read as gestational-age-adjusted multiples of the median rather than against preterm-calibrated thresholds. Second, placental pathology must be assessed and stratified using a blinded, standardised examination read independently of sFlt-1; histology can identify occult pathology only within the limits of sampling and cannot, by itself, establish that a placenta was normal, so the analysis must stratify for occult pathology rather than treat ‘normal placenta’ as a clean category. Third, informative censoring must be handled explicitly. Objective intrapartum endpoints reduce some sources of bias but do not remove it: elective early delivery can withdraw the pregnancy from labour altogether, so the fetal endpoint is itself censored by clinical action. This requires multistate or competing-events models, inverse-probability weighting, or analyses restricted to pregnancies that actually enter labour, rather than reliance on any single endpoint. Fourth, the components of the ratio must be analysed separately and judged on the appropriate endpoint: the observation that the individual components did not outperform the ratio for predicting pre-eclampsia (Zeisler et al., 2016) is not evidence against decomposition, because that endpoint is maternal and a maternal endpoint collapses the two channels the model seeks to separate. Decomposition is predicted to matter for the fetal endpoint, which that trial did not examine.

### 8.7 Latent constructs and their proxies

Because the central quantities are latent, testing depends on proxies; enumerating them is not the same as measuring the constructs, and the following matrix states what each proxy does and does not capture (**Table 1**).

## 9 Discussion: scope and limitations

The preceding sections make one argument in stages: that placental oxygen-transfer function is best defined by the balance between the fetal oxygen requirement, *C*_o_, and effective system-level supply, *O*_s_; that this balance can tip along supply-driven, demand-driven or mixed trajectories; that its clinical visibility is filtered through partly independent maternal and fetal response systems; and that a specific, delayed PlGF signal may mark it (**Table 5**). The strength of the proposal is also its principal vulnerability. Because its central quantities are latent, the framework stands or falls on whether faithful proxies can be found and whether its predictions survive the tests set out in Section 8. Several limitations bound what it can currently claim.

The framework’s central quantities are not directly measured (Section 3.1.1): the relational claim must be tested through validated proxies of fetal oxygen requirement and effective supply capacity rather than through Δ itself, and a negative proxy result constrains the model only as far as the proxies are faithful.

Oxygen is treated as the acute limiting currency most directly linked to fetal compromise, not as the exclusive metabolic determinant of placental function. Substrate availability, maternal metabolism and the placenta’s own metabolic cost may be upstream determinants or parallel axes; the model does not claim to be an account of all placental substrates, nor of the endocrine, immune and barrier roles that lie outside oxygen transfer. This is a deliberate delimitation: the framework buys sharpness on the axis most tightly coupled to acute fetal injury at the price of not explaining every metabolic dimension of placental function.

The status of the two biomarkers is asymmetric by design. The mechanistic hypothesis is developed for PlGF alone; PlGF is nonetheless not a specific sensor of fetal hypoxia, since its regulation varies with trophoblast type, gestational age, exposure duration and whether tissue expression, total circulating or free protein is measured. sFlt-1 is retained as a relevant, placentally derived biomarker but is deliberately left without a specified mechanistic role in this framework; nothing here should be read as assigning it one.

The core mechanistic sequence remains unproven as a whole. Its individual links—the placenta’s high oxygen consumption, its metabolic plasticity and the experimental downregulation of PlGF in models involving hypoxia-responsive and integrated stress-response pathways—are supported in experimental systems, but the complete biphasic sequence, including the fetal-to-placental trigger that must couple its two phases, has not been demonstrated longitudinally in an initially uncomplicated human pregnancy; its decisive staged test and the alternative explanations it must exclude are set out in Section 8.4 and not repeated here. The maternal and fetal response-modifying functions, *V*_m_ and *V*_f_, are likewise inferred rather than measured. They are conceptual modifiers whose available proxies index maternal or fetal state rather than the response functions themselves.

Finally, the clinical endpoints on which the model must be tested are imperfect. Intrapartum outcomes depend on obstetric management and are subject to informative censoring, since elective early delivery can remove labour exposure altogether; this requires the analytic safeguards described in Section 8 rather than reliance on any single endpoint. The framework also does not claim to explain every aetiology of pre-eclampsia or of fetal compromise: it proposes that a shared relational imbalance underlies an important subset of cases, not all of them. It is, in its present form, a research programme with a defined falsification strategy, not a validated clinical tool.

## 10 Conclusion

Three claims summarise the framework. First, placental sufficiency is relational: it is defined by the adequacy of effective system-level oxygen supply, *O*_s_, for the current fetal requirement, *C*_o_, not by the structure of the organ or the presence of a maternal syndrome; a positive imbalance denotes insufficiency of the integrated maternal–placental–fetal transfer relation and does not by itself identify the limiting component or imply intrinsic placental pathology, which a separate aetiological step establishes. Second, because the maternal phenotype is a host-dependent response to placental signalling that may accompany the underlying imbalance, its presence or severity cannot by itself establish fetal adequacy, and fetal compromise may develop during relative maternal silence. Third, testing the framework requires biomarker trajectories and their individual components to be related to objective fetal endpoints rather than to the maternal phenotype alone. If these predictions hold, the framework would redirect the investigation of placental disease: not only towards the mother who expresses a syndrome but also towards the fetus whose support may be failing during relative maternal silence.

## Data Availability

The original contributions presented in the study are included in the article/supplementary material. further inquiries can be directed to the corresponding author.

## References

[B1] Acharya G. Sitras V. (2009). Oxygen uptake of the human fetus at term. Acta Obstet. Gynecol Scand. 88, 104–109. doi: 10.1080/00016340802460339 18991146

[B2] Acharya G. Wilsgaard T. Rosvold Berntsen G. K. Maltau J. M. Kiserud T. (2005). Reference ranges for umbilical vein blood flow in the second half of pregnancy based on longitudinal data. Prenat. Diagn. 25, 99–111. doi: 10.1002/pd.1091 15712315

[B3] Ayres-de-Campos D. Arulkumaran S. FIGO Intrapartum Fetal Monitoring Expert Consensus Panel (2015). FIGO consensus guidelines on intrapartum fetal monitoring: physiology of fetal oxygenation and the main goals of intrapartum fetal monitoring. Int. J. Gynecol Obstet. 131, 5–8. doi: 10.1016/j.ijgo.2015.06.018 26433399

[B4] Bilginer C. Çetin A. (2026). Rethinking preeclampsia: six paradigm shifts in placental pathophysiology for maternal–foetal medicine. Front. Med. (Lausanne) 13, 1874122. doi: 10.3389/fmed.2026.1874122 42440590 PMC13333643

[B5] Bligh L. N. Greer R. M. Kumar S. (2016). The relationship between maternal *placenta*l growth factor levels and intrapartum fetal compromise. Placenta 48, 63–67. doi: 10.1016/j.placenta.2016.10.007 27871474

[B6] Braun T. Brauer M. Fuchs I. Czernik C. Dudenhausen J. W. Henrich W. . (2010). Mirror syndrome: a systematic review of fetal associated conditions, maternal presentation and perinatal outcome. Fetal Diagn. Ther. 27, 191–203. doi: 10.1159/000305096 20357423

[B7] Budak M.Ş. Toprak G. Akgöl S. Obut M. Oglak C. Baglı İ. . (2018). An investigation of the effect of placental growth factor on intrapartum fetal compromise prediction in term-induced high-risk pregnancies. Ginekol Pol. 89, 700–704. doi: 10.5603/GP.a2018.0118 30618039

[B8] Carter A. M. (2000). *Placenta*l oxygen consumption. Part I: *in vivo* studies—a review. Placenta 21, S31–S37. doi: 10.1053/plac.1999.0513 10831119

[B9] Carter A. M. (2015). Placental gas exchange and the oxygen supply to the fetus. Compr. Physiol. 5, 1381–1403. doi: 10.1002/cphy.c140073 26140722

[B10] Cavoretto P. I. Farina A. Syngelaki A. Solonos F. Nicolaides K. H. (2025). The risk for preeclampsia at 36 weeks and at 12 weeks predicts intrapartum fetal compromise and spontaneous onset of labor. Am. J. Obstet. Gynecol 233, 56–67. doi: 10.1016/j.ajog.2025.04.042 40262729

[B11] Cetin I. Taricco E. Mandò C. Radaelli T. Boito S. Nuzzo A. M. . (2020). Fetal oxygen and glucose consumption in human pregnancy complicated by fetal growth restriction. Hypertension 75, 748–754. doi: 10.1161/HYPERTENSIONAHA.119.13727 31884857

[B12] Chueh H. Y. Chang C. L. (2026). The Placental Maturation Inflection Point: an integrative biological framework for understanding early-onset versus late-onset preeclampsia. Front. Endocrinol. (Lausanne) 17, 1899767. doi: 10.3389/fendo.2026.1899767 42745947 PMC13574707

[B13] Desoye G. Wells J. C. K. (2021). Pregnancies in *diabetes* and obesity: the capacity–load model of placental adaptation. Diabetes 70, 823–830. doi: 10.2337/db20-1111 33741605 PMC7980199

[B14] Díaz P. Powell T. L. Jansson T. (2014). The role of placental nutrient sensing in maternal-fetal resource allocation. Biol. Reprod. 91, 82. doi: 10.1095/biolreprod.114.121798 25122064 PMC4435028

[B15] Enoki Y. Yasumitsu Y. Tomita S. Maeda N. Okuda T. (1972). Oxygenation properties and 2,3-diphosphoglycerate of human adult and fetal blood. Jpn. J. Physiol. 22, 557–568. doi: 10.2170/jjphysiol.22.557 4539648

[B16] Eskild A. Strøm-Roum E. M. Haavaldsen C. (2016). Does the biological response to fetal hypoxia involve angiogenesis, placental enlargement and preeclampsia? Paediatr. Perinat Epidemiol. 30, 305–309. doi: 10.1111/ppe.12283 27038011 PMC4825407

[B17] Espinoza J. Espinoza A. F. (2011). Pre-eclampsia: a maternal manifestation of a fetal adaptive response? Ultrasound Obstet. Gynecol 38, 367–370. doi: 10.1002/uog.10067 21936001

[B18] Farina A. Cavoretto P. I. Syngelaki A. Adjahou S. Nicolaides K. H. (2025). Soluble fms-like tyrosine kinase-1/placental growth factor ratio at 36 weeks’ gestation: association with spontaneous onset of labor and intrapartum fetal compromise in low-risk pregnancies. Am. J. Obstet. Gynecol 232, 392.e1–392.e14. doi: 10.1016/j.ajog.2024.08.025 39181498

[B19] Flo K. Wilsgaard T. Acharya G. (2010). Longitudinal reference ranges for umbilical vein blood flow at a free loop of the umbilical cord. Ultrasound Obstet. Gynecol 36, 567–572. doi: 10.1002/uog.7730 20586043

[B20] Fujii T. Nagamatsu T. Morita K. Schust D. J. Iriyama T. Komatsu A. . (2017). Enhanced HIF2α expression during human trophoblast differentiation into syncytiotrophoblast suppresses transcription of placental growth factor. Sci. Rep. 7, 12455. doi: 10.1038/s41598-017-12685-w 28963486 PMC5622029

[B21] Gaccioli F. Sovio U. Gong S. Cook E. Charnock-Jones D. S. Smith G. C. S. (2023). Increased placental sFLT1 drives the antiangiogenic profile of maternal serum preceding preeclampsia but not fetal growth restriction. Hypertension 80, 325–334. doi: 10.1161/HYPERTENSIONAHA.122.19482 35866422 PMC9847691

[B22] Ghi T. Fieni S. Ramirez Zegarra R. Pereira S. Dall’Asta A. Chandraharan E. (2024). Relative uteroplacental insufficiency of labor. Acta Obstet. Gynecol Scand. 103, 1910–1918. doi: 10.1111/aogs.14937 39107951 PMC11426226

[B23] Hernán M. A. Dahabreh I. J. Dickerman B. A. Swanson S. A. (2025). The target trial framework for causal inference from observational data: why and when is it helpful? Ann. Intern. Med. 178, 402–407. doi: 10.7326/ANNALS-24-01871 39961105 PMC11936718

[B24] Illsley N. P. Caniggia I. Zamudio S. (2010). Placental metabolic reprogramming: do changes in the mix of energy-generating substrates modulate fetal growth? Int. J. Dev. Biol. 54, 409–419. doi: 10.1387/ijdb.082798ni 19924633 PMC4497569

[B25] Jungelson A. Ridoux A. Barthe M. Redel D. Abbas H. Haddad B. . (2025). Total and free placental growth factor levels during preeclampsia and fetal growth restriction. Hypertension 82, 883–893. doi: 10.1161/HYPERTENSIONAHA.125.24736 40171652

[B26] Khaliq A. Dunk C. Jiang J. Shams M. Li X. F. Acevedo C. . (1999). Hypoxia down-regulates placenta growth factor, whereas fetal growth restriction up-regulates placenta growth factor expression: molecular evidence for “placental hyperoxia” in intrauterine growth restriction. Lab. Invest. 79, 151–170. 10068204

[B27] Konje J. C. Kaufmann P. Bell S. C. Taylor D. J. (2001). A longitudinal study of quantitative uterine blood flow with the use of color power angiography in appropriate for gestational age pregnancies. Am. J. Obstet. Gynecol 185, 608–613. doi: 10.1067/mob.2001.117187 11568786

[B28] Lecarpentier E. Zsengellér Z. K. Salahuddin S. Covarrubias A. E. Lo A. Haddad B. . (2020). Total versus free placental growth factor levels in the pathogenesis of preeclampsia. Hypertension 76, 875–883. doi: 10.1161/HYPERTENSIONAHA.120.15338 32654553

[B29] Link G. Clark K. E. Lang U. (2007). Umbilical blood flow during pregnancy: evidence for decreasing placental perfusion. Am. J. Obstet. Gynecol 196, 489.e1–489.e7. doi: 10.1016/j.ajog.2006.11.017 17466716

[B30] Magee L. A. Brown M. A. Hall D. R. Gupte S. Hennessy A. Karumanchi S. A. . (2022). The 2021 International Society for the Study of Hypertension in Pregnancy classification, diagnosis and management recommendations for international practice. Pregnancy Hypertens. 27, 148–169. doi: 10.1016/j.preghy.2021.09.008 35066406

[B31] Maynard S. E. Min J. Y. Merchan J. Lim K. H. Li J. Mondal S. . (2003). Excess placental soluble fms-like tyrosine kinase 1 (sFlt1) may contribute to endothelial dysfunction, hypertension, and proteinuria in preeclampsia. J. Clin. Invest. 111, 649–658. doi: 10.1172/JCI17189 12618519 PMC151901

[B32] Mizuuchi M. Cindrova-Davies T. Olovsson M. Charnock-Jones D. S. Burton G. J. Yung H. W. (2016). Placental endoplasmic reticulum stress negatively regulates transcription of placental growth factor via ATF4 and ATF6β. J. Pathol. 238, 550–561. doi: 10.1002/path.4678 26648175 PMC4784173

[B33] Nicolaides K. H. Papastefanou I. Syngelaki A. Ashoor G. Akolekar R. (2022). Predictive performance for placental dysfunction-related stillbirth of the competing risks model for small-for-gestational-age fetuses. BJOG 129, 1530–1537. doi: 10.1111/1471-0528.17066 34919332

[B34] Norman J. E. Heazell A. E. P. Rodriguez A. Weir C. J. Stock S. J. E. Calderwood C. J. . (2018). Awareness of fetal movements and care package to reduce fetal mortality (AFFIRM): a stepped wedge, cluster-randomised trial. Lancet 392, 1629–1638. doi: 10.1016/S0140-6736(18)31543-5 30269876 PMC6215771

[B35] Packet B. Van Severen R. Richter J. (2025). Vertebroplacental ratio for prediction of perinatal outcome and operative delivery for suspected fetal compromise: prospective observational cohort study. Ultrasound Obstet. Gynecol 65, 334–343. doi: 10.1002/uog.29189 39998987

[B36] Putter H. van Houwelingen H. C. (2022). Landmarking 2.0: bridging the gap between joint models and landmarking. Stat. Med. 41, 1901–1917. doi: 10.1002/sim.9336 35098578 PMC9304216

[B37] Roberts J. M. Hubel C. A. (2009). The two stage model of preeclampsia: variations on the theme. Placenta 30, S32–S37. doi: 10.1016/j.placenta.2008.11.009 19070896 PMC2680383

[B38] Rodríguez-Soto A. E. Langham M. C. Abdulmalik O. Englund E. K. Schwartz N. Wehrli F. W. (2018). MRI quantification of human fetal O2 delivery rate in the second and third trimesters of pregnancy. Magn. Reson. Med. 80, 1148–1157. doi: 10.1002/mrm.27094 29359353 PMC5980668

[B39] Saini B. S. Darby J. R. T. Portnoy S. Sun L. van Amerom J. Lock M. C. . (2020). Normal human and sheep fetal vessel oxygen saturations by T2 magnetic resonance imaging. J. Physiol. 598, 3259–3281. doi: 10.1113/JP279725 32372463

[B40] Sarno M. Wright A. Vieira N. Sapantzoglou I. Charakida M. Nicolaides K. H. (2021). Ophthalmic artery Doppler in combination with other biomarkers in prediction of pre-eclampsia at 35–37 weeks’ gestation. Ultrasound Obstet. Gynecol 57, 600–606. doi: 10.1002/uog.23517 33073902

[B41] Schabel M. C. Roberts V. H. J. Gibbins K. J. Rincon M. Gaffney J. E. Streblow A. D. . (2022). Quantitative longitudinal T2* mapping for assessing placental function and association with adverse pregnancy outcomes across gestation. PloS One 17, e0270360. doi: 10.1371/journal.pone.0270360 35853003 PMC9295947

[B42] Sherrell H. Dunn L. Clifton V. Kumar S. (2018). Systematic review of maternal placental growth factor levels in late pregnancy as a predictor of adverse intrapartum and perinatal outcomes. Eur. J. Obstet. Gynecol Reprod. Biol. 225, 26–34. doi: 10.1016/j.ejogrb.2018.03.059 29631209

[B43] Stampalija T. Monasta L. Barbieri M. Chiodo A. Quadrifoglio M. Fantasia I. . (2022). Late-term fetuses with reduced umbilical vein blood flow volume: an under-recognized population at increased risk of growth restriction. Eur. J. Obstet. Gynecol Reprod. Biol. 272, 182–187. doi: 10.1016/j.ejogrb.2022.03.032 35339807

[B44] Thadhani R. Hiemstra T. F. Vatish M. Stepan H. Cerdeira A. S. Brockelsby J. . (2026). Targeted removal of soluble Fms-like tyrosine kinase 1 in very preterm preeclampsia: a pilot trial. Nat. Med. 32, 2173–2181. doi: 10.1038/s41591-026-04333-6 42045577

[B45] Thilaganathan B. Kalafat E. (2019). Cardiovascular system in preeclampsia and beyond. Hypertension 73, 522–531. doi: 10.1161/HYPERTENSIONAHA.118.11191 30712425 PMC6380450

[B46] Turner J. M. Mitchell M. D. Kumar S. S. (2020). The physiology of intrapartum fetal compromise at term. Am. J. Obstet. Gynecol 222, 17–26. doi: 10.1016/j.ajog.2019.07.032 31351061

[B47] Wilkening R. B. Molina R. D. Meschia G. (1988). Placental oxygen transport in sheep with different hemoglobin types. Am. J. Physiol. 254, R585–R589. doi: 10.1152/ajpregu.1988.254.4.R585 3354706

[B48] Wright D. Wright A. Nicolaides K. H. (2020). The competing risk approach for prediction of preeclampsia. Am. J. Obstet. Gynecol 223, 12–23.e7. doi: 10.1016/j.ajog.2019.11.1247 31733203

[B49] Zeisler H. Llurba E. Chantraine F. Vatish M. Staff A. C. Sennström M. . (2016). Predictive value of the sFlt-1:PlGF ratio in women with suspected preeclampsia. N. Engl. J. Med. 374, 13–22. doi: 10.1056/NEJMoa1414838 26735990

